# Supplementary material for: Minimal genetically encoded tags for fluorescent protein labeling in living neurons
Source: Nat Commun. 2022 Jan 14;13:314. doi: 10.1038/s41467-022-27956-y (PMC8760255; doi:10.1038/s41467-022-27956-y)

Supplementary Figures and Supplementary Tables

Minimal genetically encoded tags for fluorescent protein labeling in living neurons

Aleksandra Arsić[1,2], Cathleen Hagemann[2], Nevena Stajković[1,2], Timm Schubert[1,3], Ivana Nikić-Spiegel[1,]*

[1] Werner Reichardt Centre for Integrative Neuroscience, University of Tübingen, Otfried-Müller-Straße 25, 72076 Tübingen, Germany

[2] Graduate Training Centre of Neuroscience, International Max Planck Research School, University of Tübingen, Otfried-Müller-Str. 27, 72074 Tübingen, Germany

[3] Institute for Ophthalmic Research, University of Tübingen, Elfriede-Aulhorn-Straße 7, 72076 Tübingen, Germany

*Corresponding Author: E-mail: ivana.nikic@cin.uni-tuebingen.de

Containing:

Supplementary Figures 1-17

Supplementary Tables 1-8

Source Data Files for western blot images

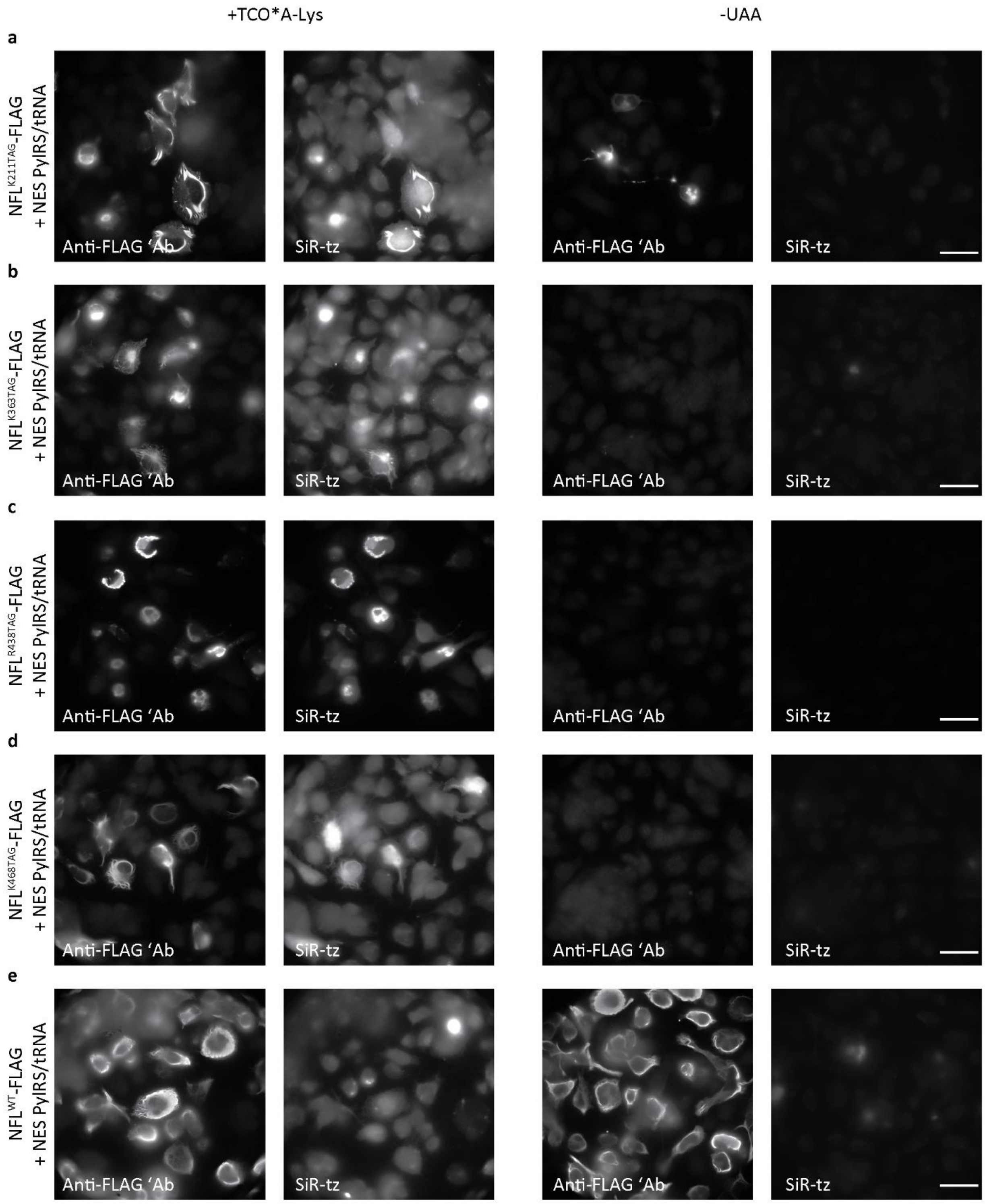

Supplementary Fig. 1 | Selection of neurofilament light chain (NFL) TAG mutants in ND7/23 cells. ND7/23 cells expressing NES PylRS/tRNA$_{CUA}^{Pyl}$, NFM, and NFL^{K211TAG}-FLAG (**a**), NFL^{K363TAG}-FLAG (**b**), NFL^{R438TAG}-FLAG (**c**), NFL^{K468TAG}-FLAG (**d**) or NFL^{WT}-FLAG (**e**). After incubation overnight with or without TCO*A-Lys, cells were labeled with SiR-tetrazine (SiR-tz), fixed and stained with anti-FLAG antibody,

followed by Alexa Fluor (AF) 488-conjugated secondary antibody. Images were acquired with widefield microscopy. Data were collected from three independent experiments. Scale bars: 50 µm (**a**–**e**).

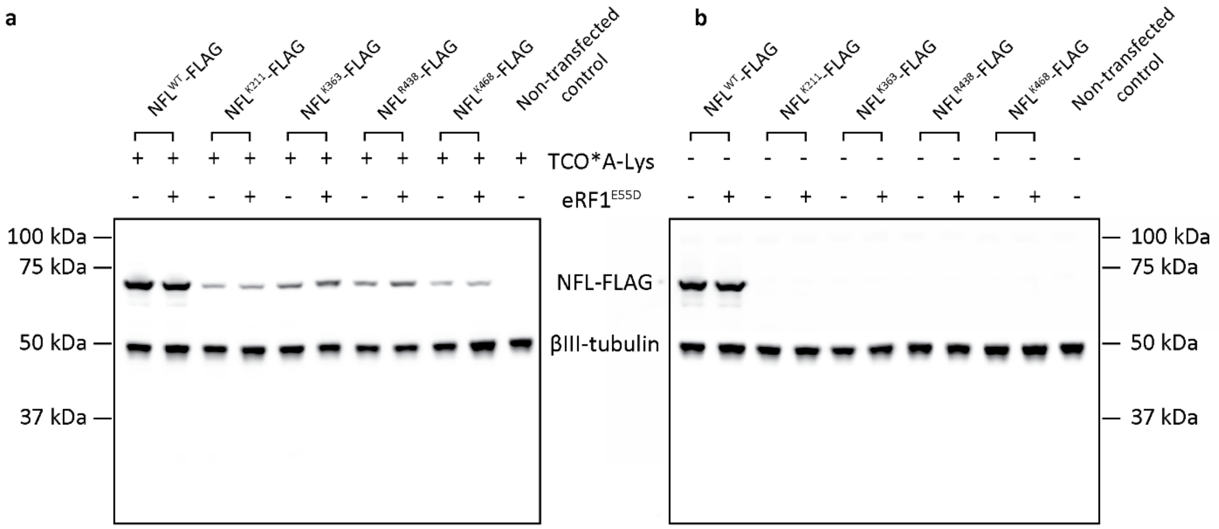

c

sample	NFL^{WT}-FLAG		NFL^{K211}-FLAG		NFL^{K363}-FLAG		NFL^{R438}-FLAG		NFL^{K468}-FLAG	
eRF1^{E55D}	-	+	-	+	-	+	-	+	-	+
% of the WT	100%	82.2%	29.4%	37.9%	39.8%	49.2%	35.6%	46.9%	24.6%	28%
SEM	0	3.4	2.7	2.6	1.8	3.7	3.5	4.9	1.7	5.3

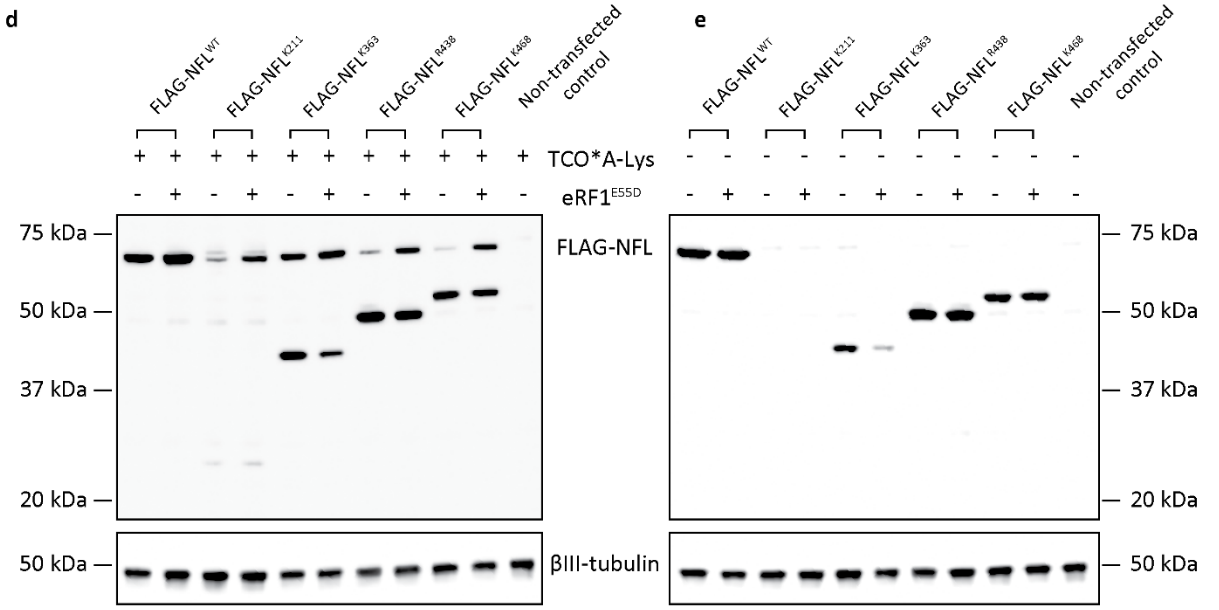

f

sample	FLAG-NFL^{K211}		FLAG-NFL^{K363}		FLAG-NFL^{R438}		FLAG-NFL^{K468}	
eRF1^{E55D}	-	+	-	+	-	+	-	+
Full-length NFL % of the total NFL^{TAG}	96.4%	98.4%	35.2%	79.9%	3.2%	23.5%	2.5%	25.2%
NFL fragment % of the total NFL^{TAG}	3.6%	1.6%	64.8%	20.1%	96.8%	76.5%	97.5%	74.8%

Supplementary Fig. 2 | Western blot analysis of NFL WT and TAG mutant expression levels in ND7/23 cells. ND7/23 cells were transfected with NFL^{WT}-FLAG and NFL^{TAG}-FLAG mutants (**a-c**), or with FLAG-NFL^{WT} and FLAG-NFL^{TAG} mutants (**d-f**), with NES PylRS/$tRNA_{CUA}^{Pyl}$, NFM, and either empty pcDNA3.1/Zeo(+) or pcDNA3.1/Zeo(+) containing eukaryotic release factor 1 mutant E55D ($eRF1^{E55D}$) encoding sequence. After incubation overnight with (**a,d**) or without (**b,e**) TCO*A-Lys, cells were lysed and lysates were analyzed by western blot. Proteins were labeled with anti-FLAG and anti-βIII-tubulin antibodies. **c**, Quantitative analysis of NFL-FLAG expression levels based on the anti-FLAG band intensity, normalized to the anti-βIII-tubulin band intensity, shown as percentage of the NFL^{WT} expression. The table shows average values from three independent experiments and corresponding standard error of the mean (SEM). **f**, Analysis of the relative amount of full-length and truncated FLAG-NFL^{TAG} proteins compared to the total amount of the expressed FLAG-NFL^{TAG}. The table shows average values from three independent experiments. Full data sets collected across three experiments, corresponding to the tables in **c,f** are shown in Supplementary Table 1 and Supplementary Table 2, respectively. Source data are provided as a Source Data file.

a

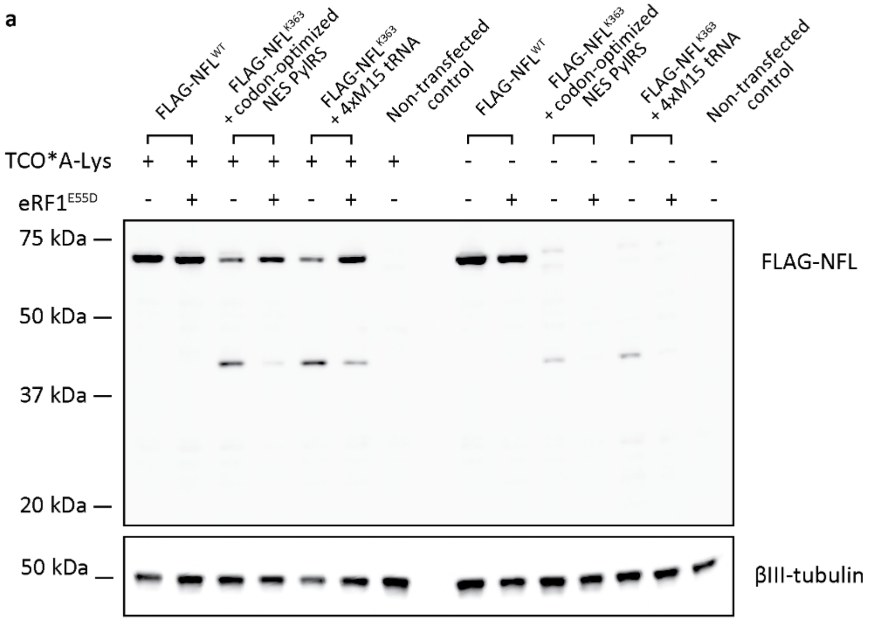

b

sample	FLAG-NFL^{K363} + codon-optimized NES PylRS		FLAG-NFL^{K363} + 4xM15 tRNA	
eRF1^{E55D}	-	+	-	+
Full-length NFL % of the total NFL^{K363TAG}	48.6%	97.4%	40.4%	90.1%
NFL fragment % of the total NFL^{K363TAG}	51.4%	2.6%	59.6%	9.9%

Supplementary Fig. 3 | Western blot analysis of the ratio between full-length and truncated NFL^{K363TAG} after expression with improved amber codon suppression constructs. a, ND7/23 cells were transfected with FLAG-NFL^{WT} or FLAG-NFL^{K363TAG}, NFM, empty pcDNA3.1/Zeo(+) or pcDNA3.1/Zeo(+)-eRF1^{E55D}, as well as with a construct containing either codon-optimized NES PylRS/tRNA$_{CUA}^{Pyl}$ or NES PylRS/4xM15tRNA$_{CUA}$. After incubation overnight with or without TCO*A-Lys, cells were lysed and lysates were analyzed by western blot. Proteins were labeled with anti-FLAG and anti-βIII-tubulin antibodies. **b**, Analysis of the relative amount of full-length and truncated FLAG-NFL^{K363TAG} proteins compared to the total amount of the expressed FLAG-NFL^{K363TAG}. Representative data from three independent experiments. Source data are provided as a Source Data file.

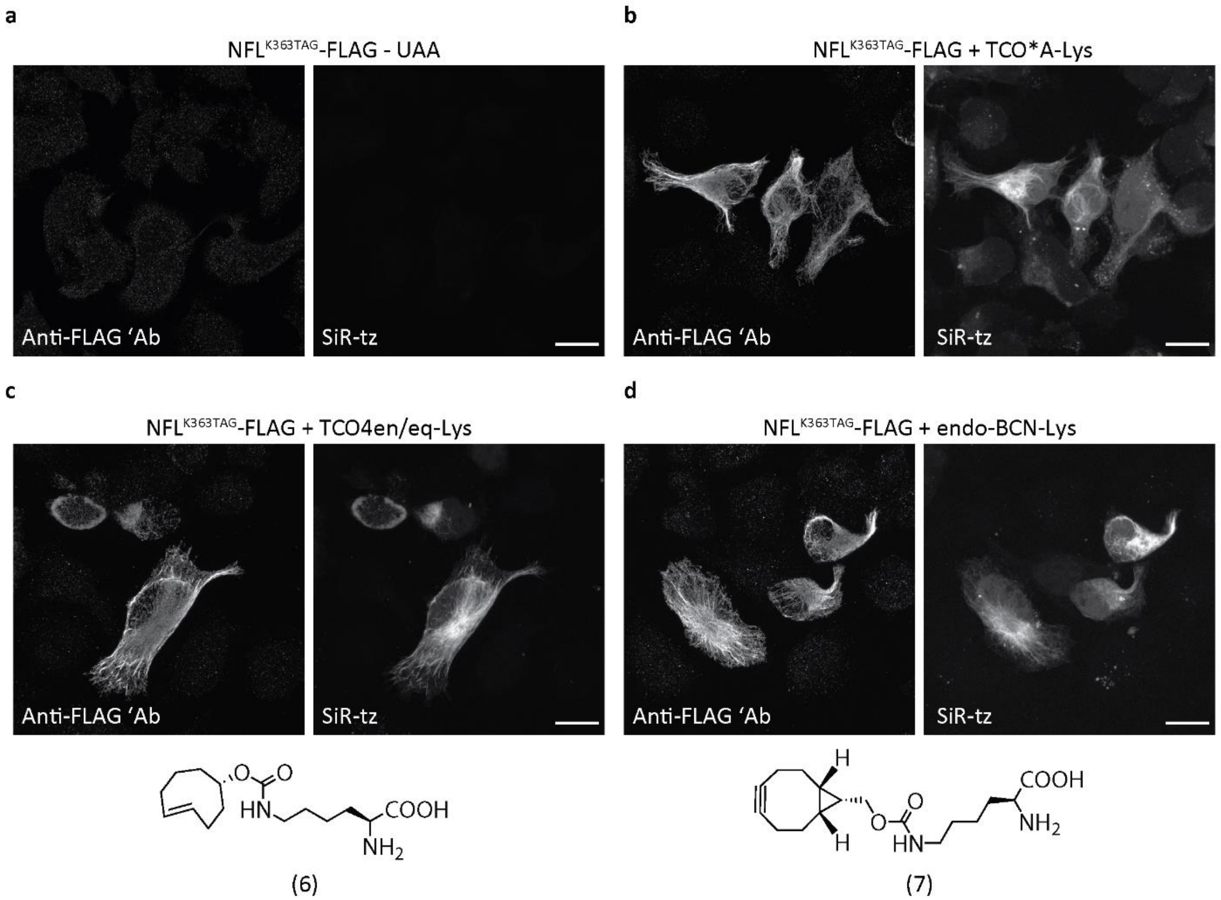

Supplementary Fig. 4 | Expression of the selected NFL^{K363TAG}-FLAG mutant with different unnatural amino acids (UAAs). ND7/23 cells were transfected with NFL^{K363TAG}-FLAG, NFM, and NES PylRS/tRNA$_{CUA}^{Pyl}$ constructs and incubated overnight, either without UAA (**a**), or with TCO*A-Lys (**b**), TCO4en/eq-Lys (**c**), endo-BCN-Lys (**d**). Chemical structures of TCO4en/eq-Lys (6) and endo-BCN-Lys (7) are shown below the corresponding panels. Chemical structure of TCO*A-Lys is shown in the Fig. 1. Cells were labeled with SiR-tz, fixed and stained with anti-FLAG antibody, followed by AF488-conjugated secondary antibody. Images were acquired as a single plane (**a**) or as *Z*-stacks on a confocal scanning microscope (**b**–**d**). *Z*-stack images are shown as maximum intensity projections (**b**–**d**). Data were collected from three independent experiments. Scale bars: 20 µm (**a**–**d**).

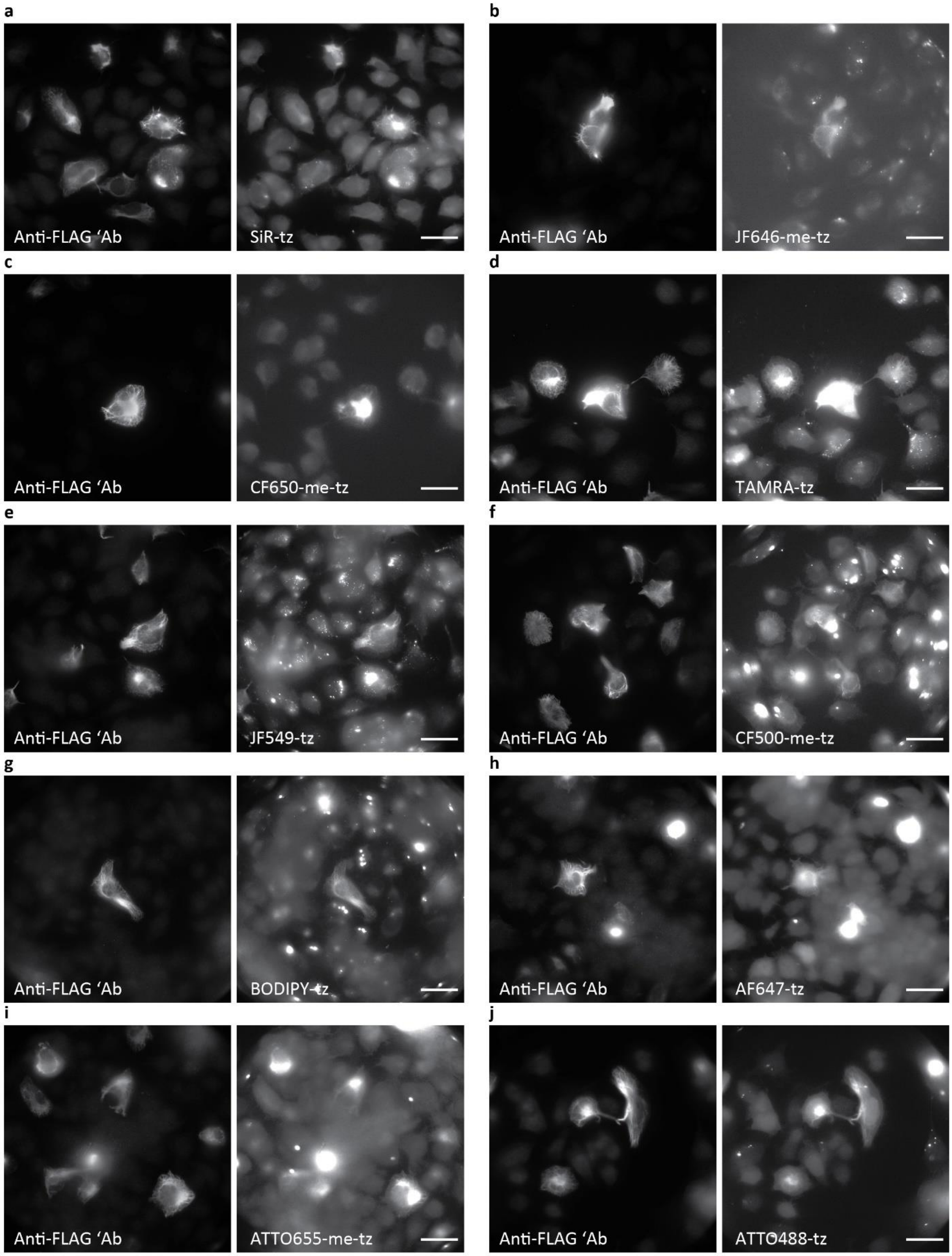

Supplementary Fig. 5 | Labeling of NFL^{K363TAG}-FLAG mutant with various cell-permeable and cell-impermeable tetrazine dyes in ND7/23 cells. ND7/23 cells expressing NFL^{K363TAG}-FLAG, NFM, and NES

PylRS/tRNA$_{CUA}^{Pyl}$ constructs. **a**–**g**, After incubation overnight with TCO*A-Lys, living cells were labeled with SiR-tz (**a**), JF646-me-tz (**b**), CF650-me-tz (**c**), TAMRA-tz (**d**), JF549-tz (**e**), CF500-me-tz (**f**), or BODIPY-tz (**g**), then fixed and stained with anti-FLAG antibody. **h**–**j**, After incubation overnight with TCO*A-Lys, cells were fixed and stained with AF647-tz (**h**), ATTO655-me-tz (**i**) or ATTO488-tz (**j**), and with anti-FLAG antibody. Secondary antibodies used for FLAG labeling were conjugated with AF488 (**a**–**e**, **h** and **i**) or AF647 (**f**,**g**,**j**). Images were acquired with widefield microscopy. Data were collected from three independent experiments. Scale bars: 50 µm (**a**–**j**).

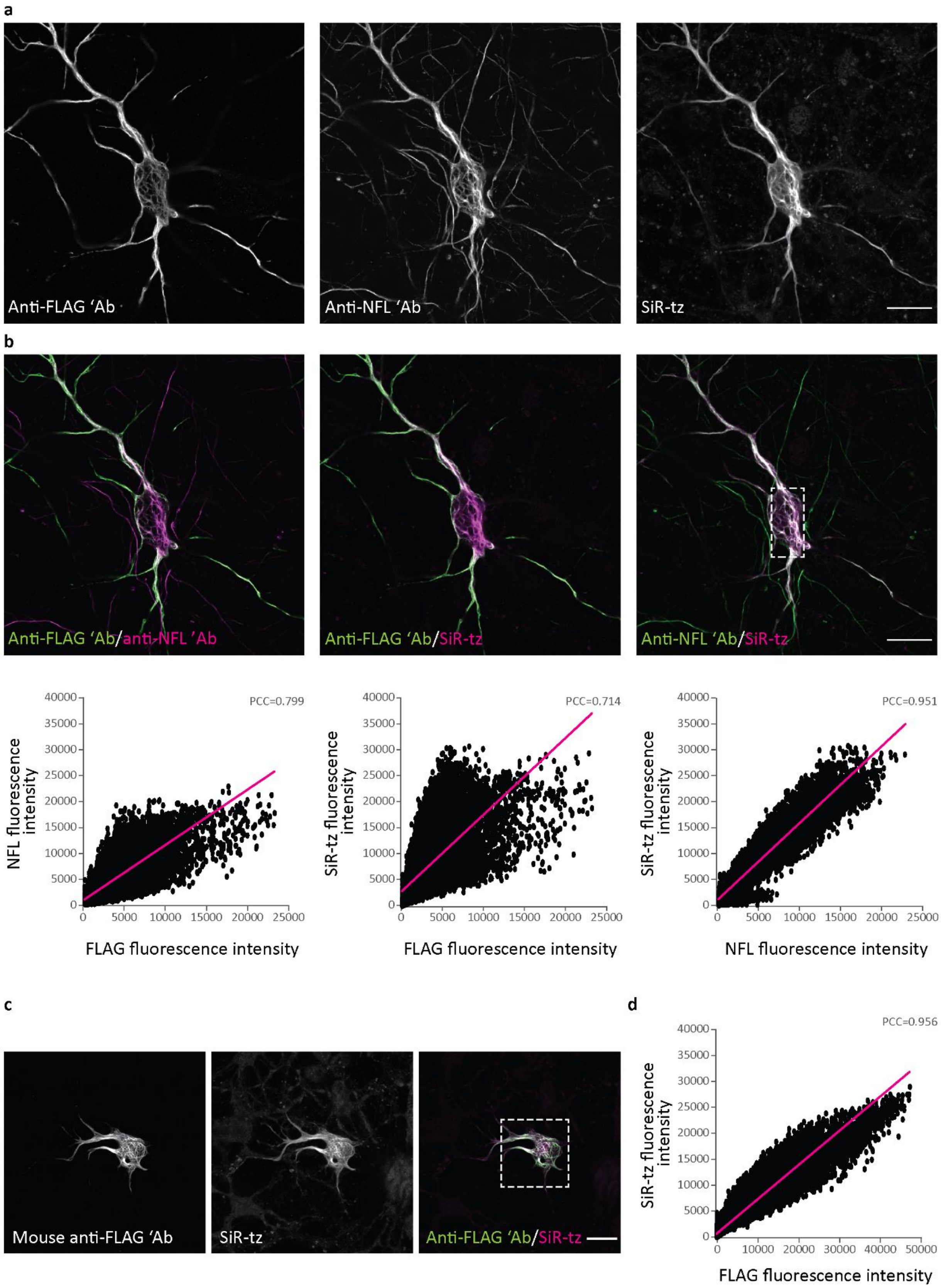
a
Anti-FLAG 'Ab
Anti-NFL 'Ab
SiR-tz
b
Anti-FLAG 'Ab/anti-NFL 'Ab
Anti-FLAG 'Ab/SiR-tz
Anti-NFL 'Ab/SiR-tz
PCC=0.799
PCC=0.714
PCC=0.951
NFL fluorescence intensity
SiR-tz fluorescence intensity
FLAG fluorescence intensity
NFL fluorescence intensity
c
Mouse anti-FLAG 'Ab
SiR-tz
Anti-FLAG 'Ab/SiR-tz
d
PCC=0.956
SiR-tz fluorescence intensity
FLAG fluorescence intensity

Supplementary Fig. 6 | Colocalization analysis of NFL^{K363TAG}-FLAG labeling with anti-FLAG, anti-NFL antibody, and click chemistry. a−**c**, MCNs were transfected with NFL^{K363TAG}-FLAG, NFM, and NES PylRS/tRNA$_{CUA}^{Pyl}$ constructs, incubated for 3 days with TCO*A-Lys and labeled with SiR-tz. After 2−3 h of washing, MCNs were fixed and stained with either rabbit anti-FLAG (**a**,**b**) or with mouse anti-FLAG antibody (**c**). Single-plane images were acquired on a confocal scanning microscope. **a**, Neuron expressing NFL^{K363TAG}-FLAG, labeled with rabbit anti-FLAG antibody, anti-NFL antibody, and SiR-tz. **b**, Merge images showing the colocalization of the three channels, with the corresponding colocalization scatterplots below each image. Colocalization analysis was performed in the region outlined by the dashed box, to avoid NFL signal from the surrounding, non-transfected neurons. Pearson's correlation coefficients (PCC) are shown above each scatterplot. **c**, Since the colocalization of SiR-tz and anti-FLAG antibody labeling was not as high as we expected, we performed immunolabeling of NFL^{K363TAG}-FLAG with an additional, mouse anti-FLAG antibody. Colocalization analysis was performed in the region outlined by the dashed box. **d**, Scatterplot showing the colocalization of mouse anti-FLAG antibody with SiR-tz labeling from (**c**), and the corresponding Pearson's correlation coefficient. Fluorescence intensities in graphs are shown as absolute grey values of 16-bit depth images (**b,d**). Data were collected from three (**a,b**) or two (**c,d**) independent experiments. Source data are provided as a Source Data file. Scale bars 20 µm (**a**–**c**).

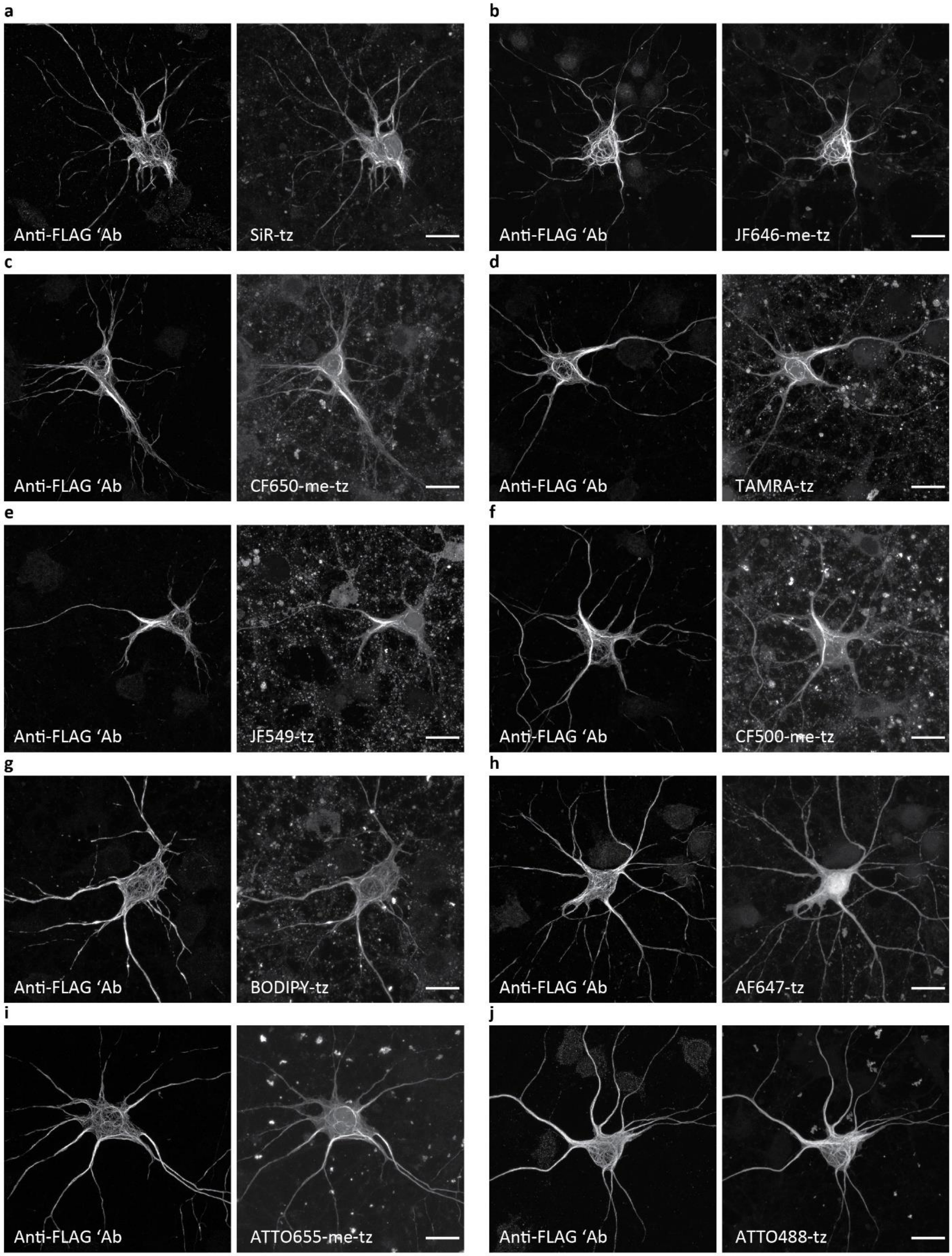

Supplementary Fig. 7 | Labeling of NFL^{K363TAG}-FLAG mutant with various cell-permeable and cell-

impermeable tetrazine dyes in primary mouse cortical neurons (MCNs). MCNs expressing NFL^{K363TAG}-FLAG, NFM, and NES PylRS/tRNA$_{CUA}^{Pyl}$ constructs. **a**–**g**, After incubation for 2–3 days in the presence of TCO*A-Lys, living neurons were labeled with SiR-tz (**a**), JF646-me-tz (**b**), CF650-me-tz (**c**), TAMRA-tz (**d**), JF549-tz (**e**), CF500-me-tz (**f**), or BODIPY-tz (**g**), then fixed and stained with anti-FLAG antibody. **h**–**j**, After incubation for 2–3 days in the presence of TCO*A-Lys, neurons were fixed and stained with AF647-tz (**h**), ATTO655-me-tz (**i**) or ATTO488-tz (**j**), and with anti-FLAG antibody. Secondary antibodies used for FLAG labeling were conjugated with AF488 (**a-e**, **h** and **i**), AF647 (**f**,**g**) or AF555 (**j**). *Z*-stack images were acquired on a confocal scanning microscope and are shown as maximum intensity projections. Data were collected from three independent experiments. Scale bars: 20 µm (**a**–**j**).

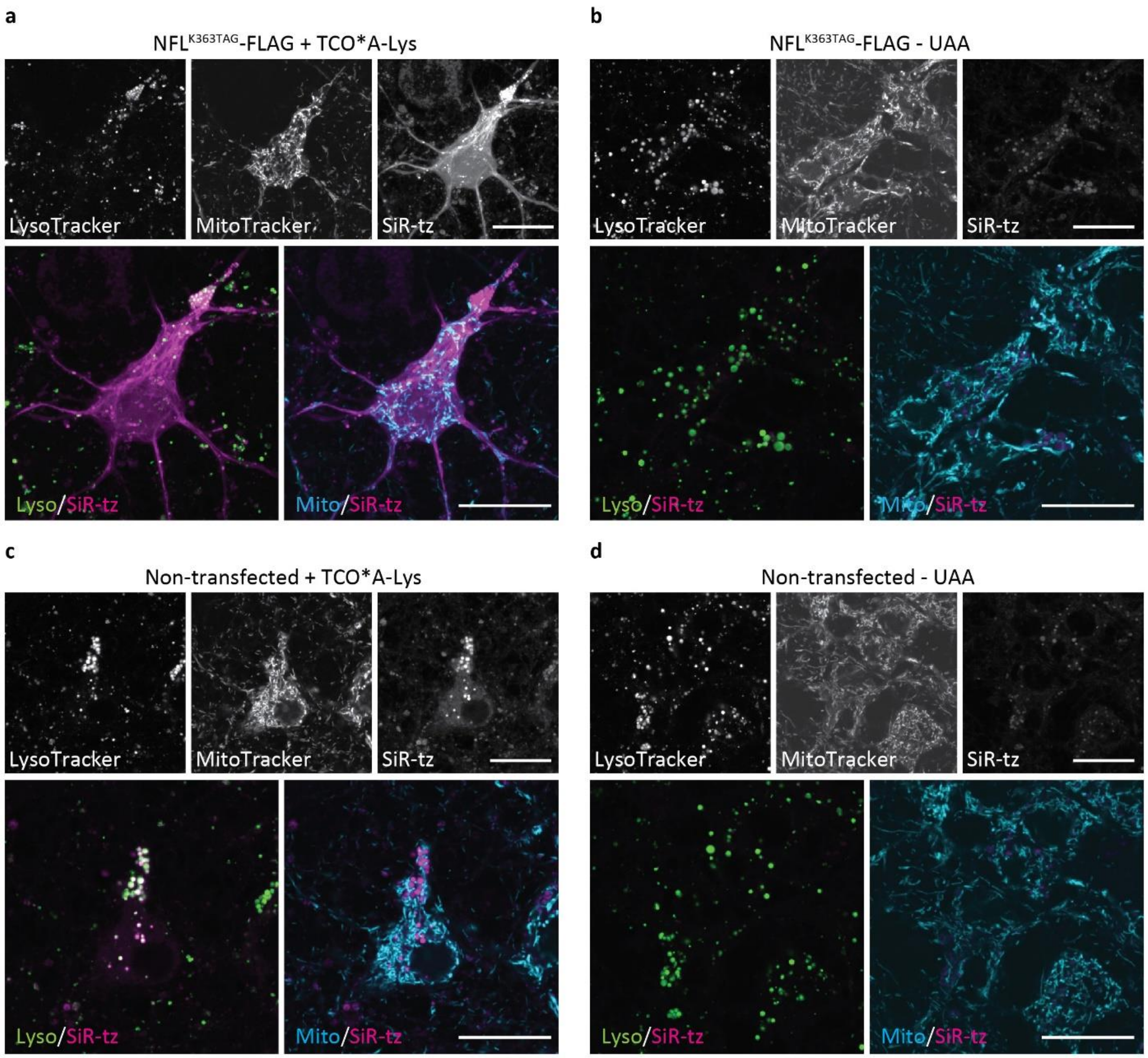

Supplementary Fig. 8 | Accumulation of tetrazine dyes in lysosomes in the presence of UAA. Primary mouse cortical neurons were transfected with $NFL^{K363TAG}$-FLAG, NFM, and NES PylRS/$tRNA_{CUA}^{Pyl}$ constructs (**a**,**b**) or with no DNA-containing sham (**c**,**d**). After 3 days of incubation with (**a**,**c**) or without (**b**,**d**) TCO*A-Lys, neurons were labeled with SiR-tz, MitoTracker Orange and LysoTracker Green, and imaged live on a confocal scanning microscope. Brightness and contrast were linearly adjusted to show the same display range in SiR-tz panels, while other panels were adjusted individually for optimal visualization. Data were collected from three independent experiments. Scale bars: 20 µm (**a**–**d**).

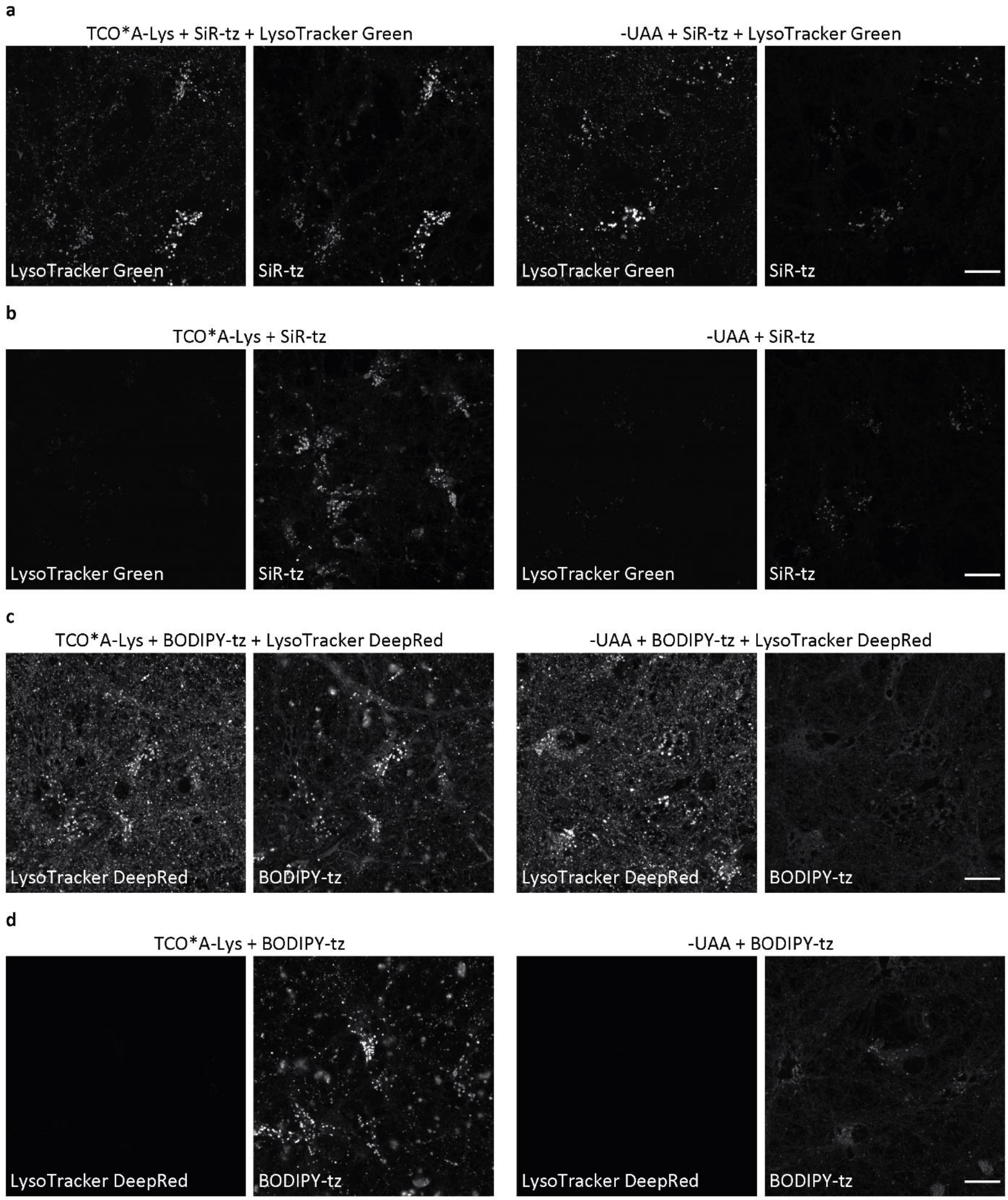

Supplementary Fig. 9 | Accumulation of tetrazine dyes in lysosomes does not depend on the presence of LysoTracker dyes. a–**d**, MCNs were incubated with TCO*A-Lys or without UAA for 3 days, and labeled with either SiR-tz (**a**,**b**) or BODIPY-tz (**c**,**d**). After 3 h of washing, MCNs were labeled with LysoTracker Green

(**a**), LysoTracker DeepRed (**c**) or were not labeled (**b**,**d**), and were imaged live on a confocal scanning microscope. Data were collected from three independent experiments. Scale bars: 20 µm (**a**–**d**).

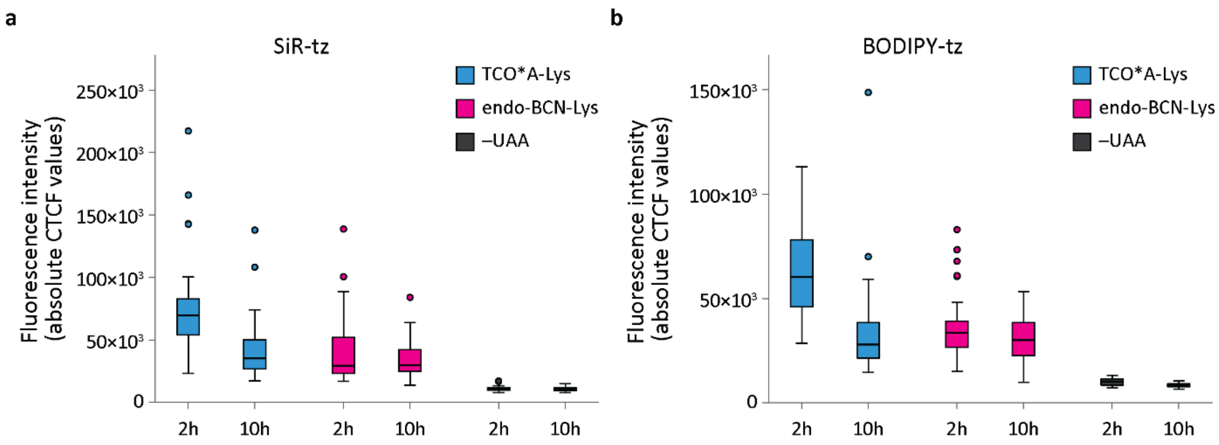

Supplementary Fig. 10 | Quantification of the click chemistry labeling background fluorescence intensity. a–b, MCNs were transfected with NFL^{K363TAG}-FLAG, NFM, and NES PylRS/tRNA$_{CUA}^{Pyl}$ constructs and incubated for 2 days with TCO*A-Lys, endo-BCN-Lys or without UAA. Afterwards, neurons were labeled with either SiR-tz (**a**) or BODIPY-tz (**b**) and fixed after washing for 2 h or 10 h. After fixation, neurons were labeled with anti-FLAG primary antibody, followed by AF488-conjugated (**a**) or AF647-conjugated (**b**) secondary antibody and imaged on the confocal scanning microscope. Intensity of the click chemistry labeling background was measured in Fiji and used for the calculation of CTCF (calculated total cell fluorescence intensity) values. Data were collected from three independent experiments, with n = 30 images per condition. Kruskal-Wallis non-parametric test, followed by a Mann-Whitney U test for pairwise comparisons (with Bonferroni correction for multiple comparisons) were run to compare the CTCF values of different groups. Detailed results of this statistical analysis can be found in Supplementary Tables 6 and 7. The top and bottom box lines show first and third quartile of the data, while the line encompassed by the box represents the data median. Boxplot whiskers show the minimum and maximum values, with circles representing outliers. Source data are provided as a Source Data file.

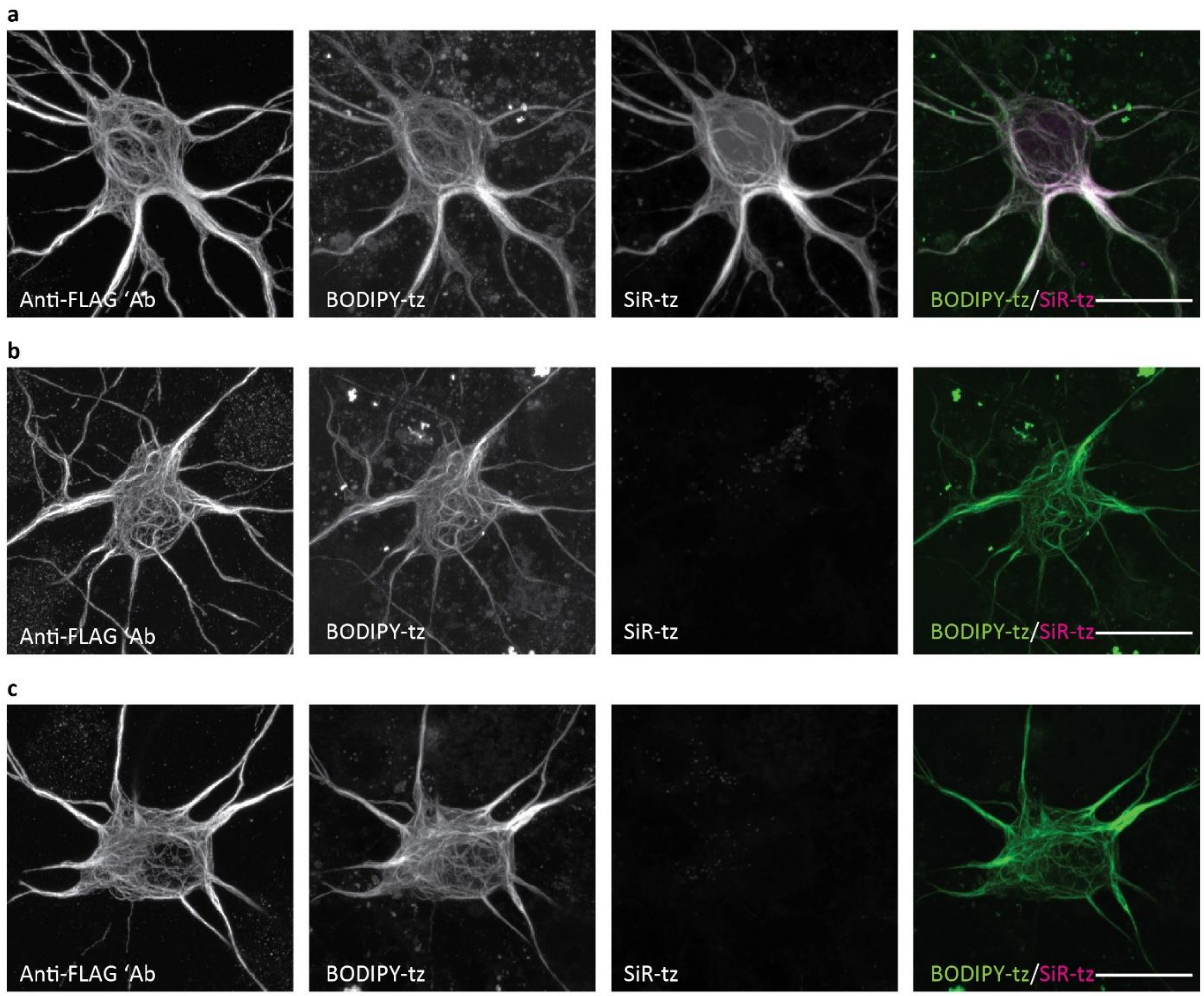

Supplementary Fig. 11 | Controls for pulse-chase click labeling of two NFL populations in primary mouse cortical neurons (MCNs). **a**–**d**, MCNs were transfected with $NFL^{K363TAG}$-FLAG, NFM, and NES PylRS/$tRNA_{CUA}^{Pyl}$ constructs, incubated for 2 days with TCO*A-Lys and labeled with BODIPY-tz dye. After the labeling, neurons were incubated for a further 2 days with TCO*A-Lys (**a**), or without TCO*A-Lys (**b**) and labeled with SiR-tz dye. **c**, Neurons were labeled with SiR-tz immediately after BODIPY-tz labeling. After the second labeling, neurons were fixed and stained with anti-FLAG antibody, followed by AF555-conjugated secondary antibody. *Z*-stack images were acquired on a confocal scanning microscope and are shown as maximum intensity projections. Data were collected from three independent experiments. Scale bars: 20 µm (**a**–**c**).

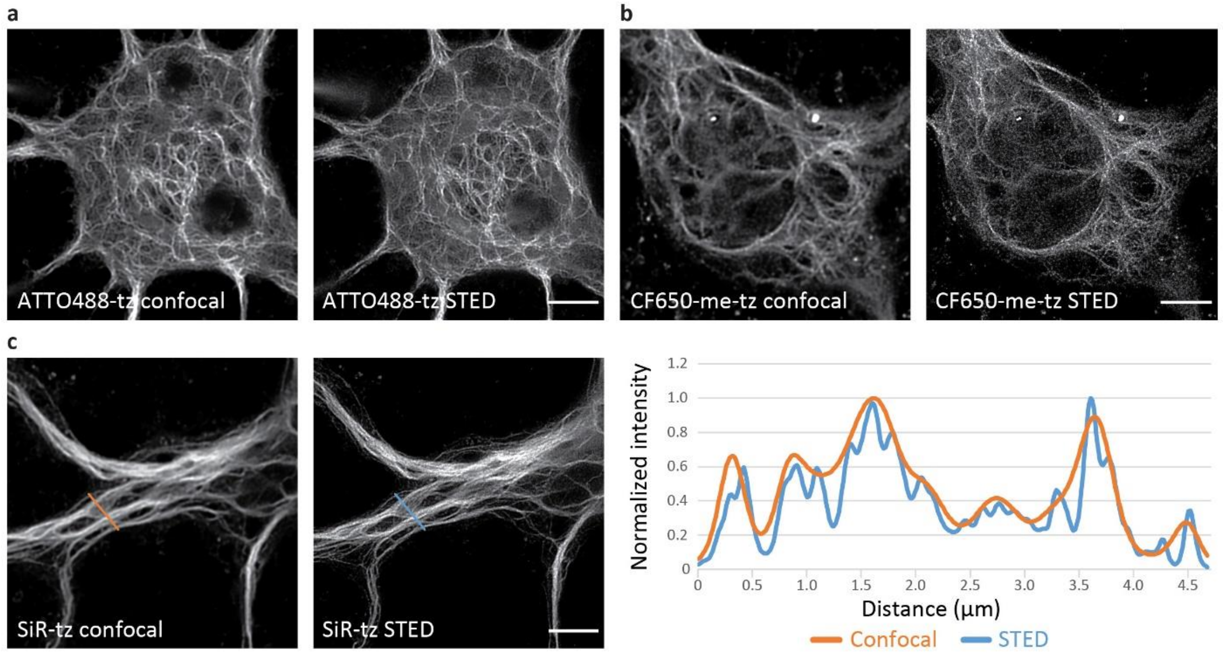

Supplementary Fig. 12 | Additional examples of stimulated emission depletion (STED) imaging of NFL in primary mouse cortical neurons (MCNs). a–**c**, MCNs expressing $NFL^{K363TAG}$-FLAG, NFM, and NES PylRS/$tRNA_{CUA}^{Pyl}$ in the presence of TCO*A-Lys. After 2–3 days of expression, NFL was labeled with ATTO488-tz (**a**), CF650-me-tz (**b**) or SiR-tz (**c**) and imaged with STED microscopy. The line profile graph in panel **c** demonstrates the increase in resolution of STED image (blue line) in comparison to the confocal image (orange line). Raw confocal and STED images were deconvolved using Huygens deconvolution software. Data were collected from three independent experiments. Source data are provided as a Source Data file. Scale bars: 5 µm (**a**–**c**).

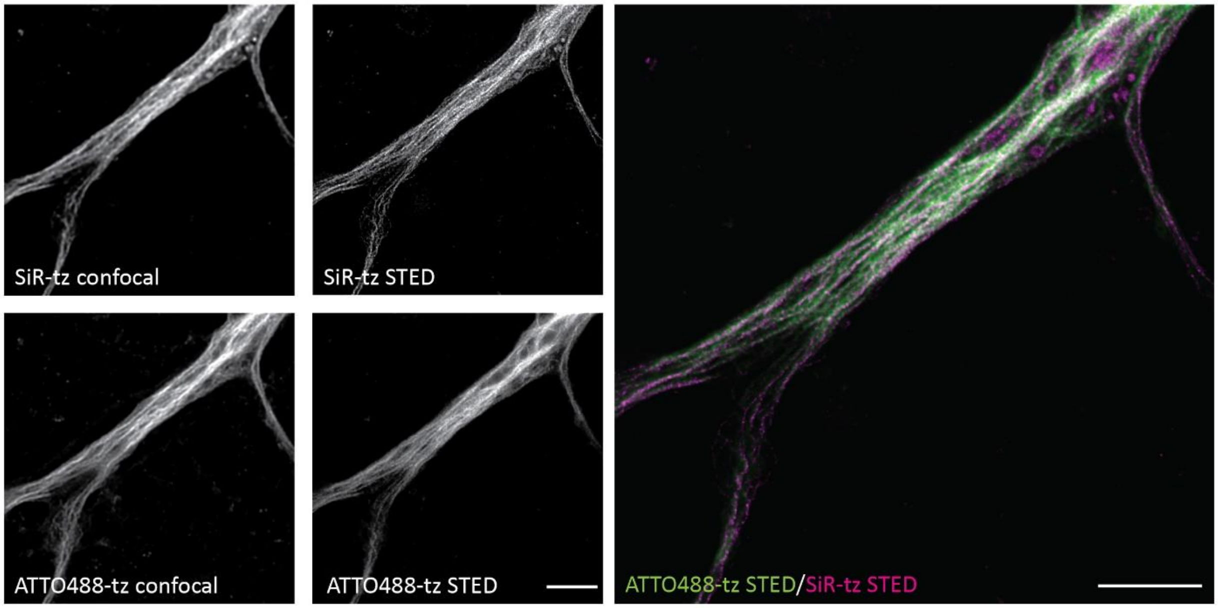

Supplementary Fig. 13 | Additional examples of STED imaging of two populations of click-labeled NFL in primary mouse cortical neurons (MCNs). MCNs expressing NFL^{K363TAG}-FLAG, NFM, and NES PylRS/tRNA$_{CUA}^{Pyl}$ in the presence of TCO*A-Lys. After 2 days of expression, neurons were labeled with SiR-tz, incubated for a further two days with TCO*A-Lys, and labeled after fixation with ATTO488-tz. Click-labeled NFL was imaged with STED microscopy. Raw confocal and STED images were deconvolved using Huygens deconvolution software. Data were collected from three independent experiments. Scale bars: 5 µm.

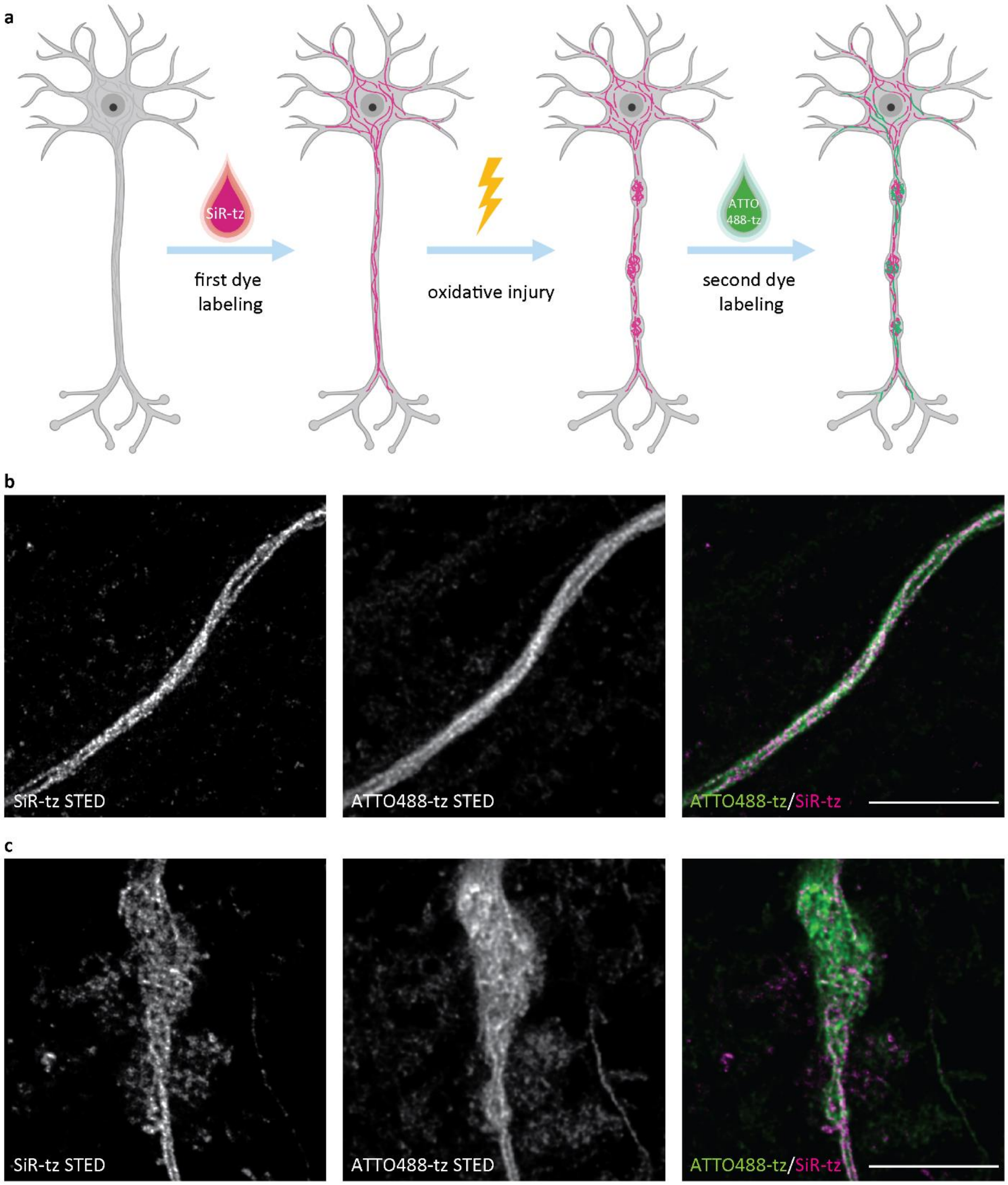

Supplementary Fig. 14 | Pulse–chase click labeling of two NFL populations can be used in combination with STED imaging for tracking NFL distribution during oxidative injury. a, A schematic representation of the experimental design. MCNs were transfected with NFLK363TAG-FLAG, NFM, and NES PylRS/tRNA$_{CUA}^{Pyl}$ constructs. After incubation with TCO*A-Lys for 2 days, NFL was labeled with SiR-tz, and neurons were subjected to nitric-oxide-mediated oxidative injury. After injury, neurons were incubated with TCO*A-Lys

for a further 2 days, fixed, and stained with ATTO488-tz. **b**, STED images of a healthy axon with two populations of click-labeled NFL. **c**, STED images of an injured axon with two populations of click-labeled NFL. STED images were deconvolved using Huygens deconvolution software. Data were collected from three independent experiments. Scale bars: 5 µm (**b**,**c**).

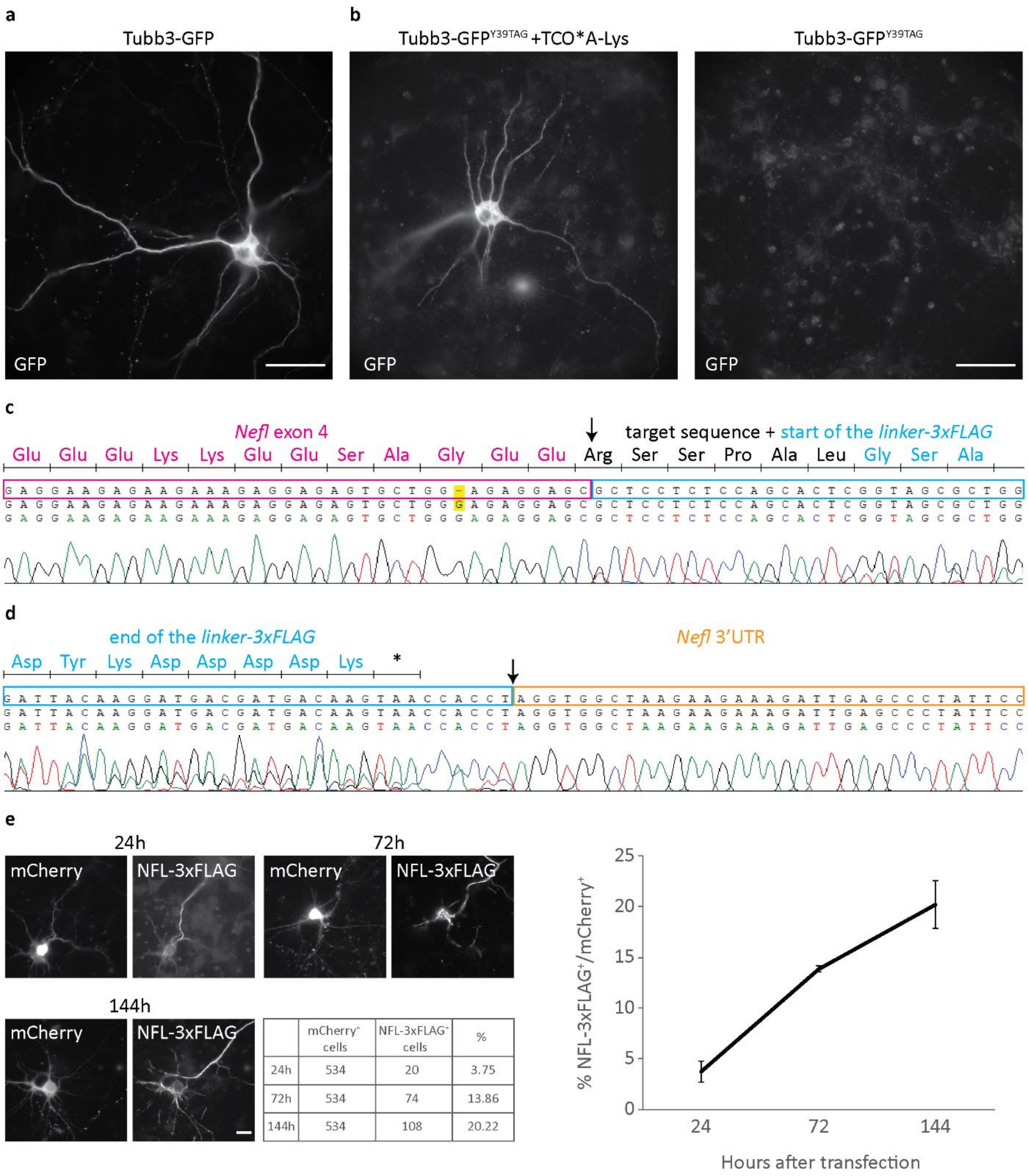

	mCherry+ cells	NFL-3xFLAG+ cells	%
24h	534	20	3.75
72h	534	74	13.86
144h	534	108	20.22

Supplementary Fig. 15 | Optimization of endogenous protein labeling via ORANGE knock-in (KI), amber codon suppression and click chemistry. a−b, Optimization of amber codon suppression of endogenous βIII tubulin (Tubb3) in primary mouse cortical neurons (MCNs). **a**, MCNs were transfected with the pORANGE Tubb3-GFP KI construct, together with the NES PylRS/$tRNA_{CUA}^{Pyl}$ and $eRF1^{E55D}$ at day *in vitro* (DIV) 3. After 6 days of expression, neurons were fixed and endogenous Tubb3 tagged with GFP was

imaged with widefield microscopy. **b**, MCNs were transfected with the pORANGE Tubb3 GFP^{Y39TAG} KI construct, together with the NES PylRS/$tRNA_{CUA}^{Pyl}$ and $eRF1^{E55D}$. After incubation for 6 days with or without TCO*A-Lys, neurons were fixed and endogenous Tubb3 tagged with $GFP^{Y39\rightarrow UAA}$ was imaged with widefield microscopy. Data were collected from three independent experiments. **c-d**, Analysis of ORANGE-mediated knock-in specificity. MCNs were transfected at DIV5 with pORANGE NFL $linker^{WT}$-3xFLAG KI. After 4 days, genomic DNA was collected and used as a template for the amplification of 5' and 3' donor integration junctions. Sequencing of the 5' (**c**) and 3' (**d**) junction PCR products confirmed that the donor DNA was integrated correctly into the genomic DNA. Arrows in **c-d** annotate junction positions. Note that the template for junction PCR is a mixture of genomic DNA sequences which are collected from MCNs. For this reason, sequencing results show a single nucleotide insertion (marked in yellow, **c**), which is a common consequence of genome editing and is not present in all genomic sequences. Another consequence of using a mixture of genomic DNA sequences are multiple chromatogram peaks which are present in the 3xFLAG sequence. **e**, Quantification of ORANGE-mediated knock-in efficiency. MCNs were transfected at DIV5 with pORANGE NFL $linker^{WT}$-3xFLAG KI and pcDNA3.1/Zeo(+)-mCherry constructs. After incubation for 24 h, 72 h and 144 h, neurons were fixed and stained with anti-FLAG antibody, followed by AF488-conjugated secondary antibody. Panels show representative widefield images of $mCherry^+$ and NFL-$3xFLAG^+$ cells that were used for quantification. Numbers of $mCherry^+$ and NFL-$3xFLAG^+$ cells collected across three experiments, as well as the percentage of NFL-$3xFLAG^+$/$mCherry^+$ cells are shown in the table. The corresponding graph on the left shows the mean value with standard error of the mean for each time point. Source data are provided as a Source Data file. Scale bars: 50 µm (**a**,**b**), 20 µm (**e**).

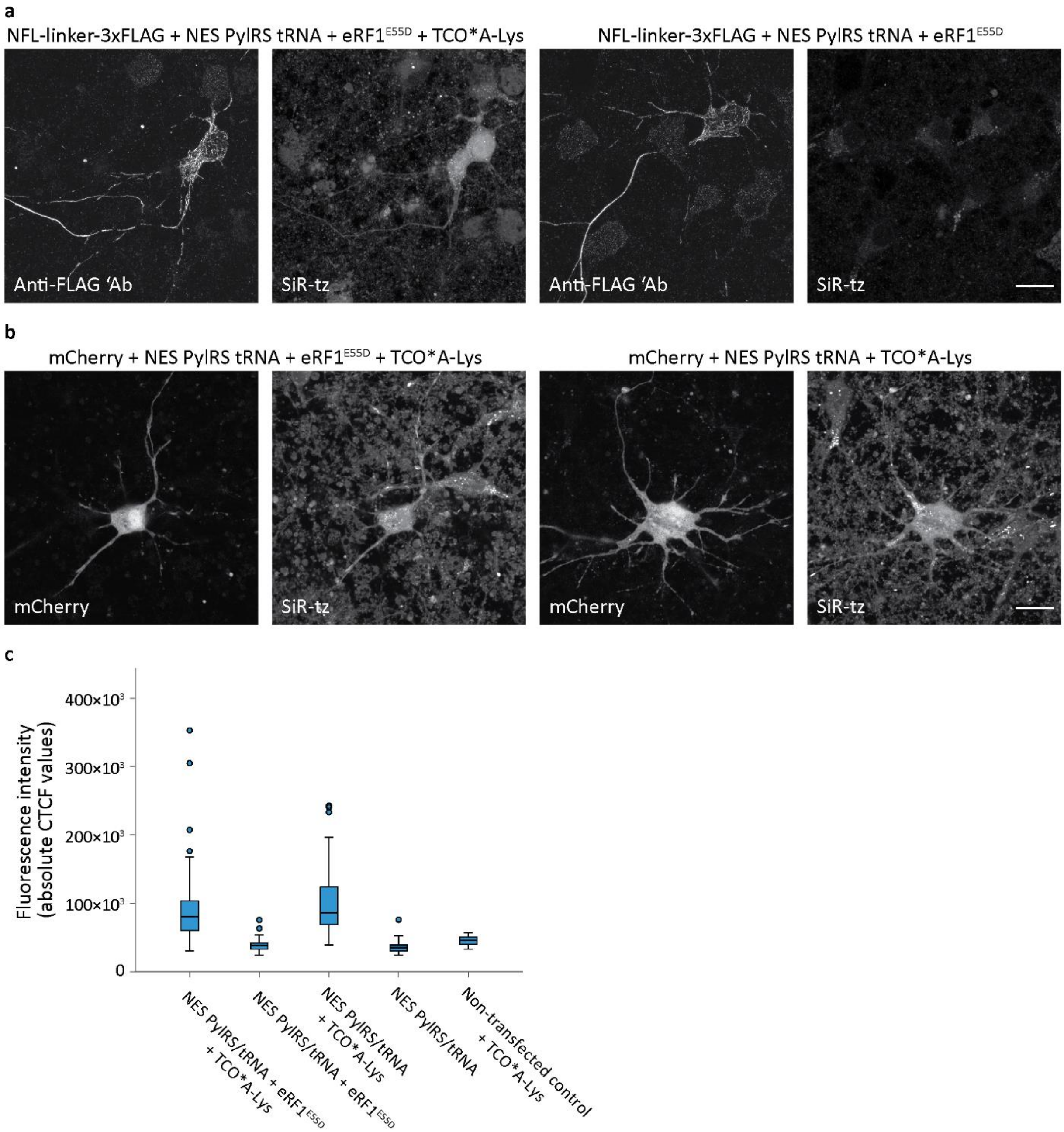

Supplementary Fig. 16 | Additional controls of nonspecific click labeling background in MCN. a, MCNs were transfected with pORANGE NFL linker^{WT}-3xFLAG KI, NES PylRS/tRNA$_{CUA}^{Pyl}$ and eRF1^{E55D} at DIV5. After incubation for 6 days with or without TCO*A-Lys, neurons were labeled with SiR-tz, fixed and stained with anti-FLAG antibody, followed by AF488-conjugated secondary antibody. *Z*-stack images were acquired on a confocal scanning microscope and are shown as maximum intensity projections. Data were collected from three independent experiments. **b**, MCNs were transfected with pcDNA3.1/Zeo(+)-mCherry, NES PylRS/tRNA$_{CUA}^{Pyl}$, with or without eRF1^{E55D} at DIV5. After incubation for 6 days with TCO*A-Lys, neurons

were labeled with SiR-tz and fixed. Single-plane images were acquired on a confocal scanning microscope. Data were collected from three independent experiments. **c**, Quantification of the click chemistry labeling background fluorescence intensity in the presence or absence of eRF1^{E55D}. MCNs were transfected, labeled, and imaged as described in **b**. Intensity of the click labeling background was measured in Fiji and used for the calculation of CTCF (calculated total cell fluorescence intensity) values. Data were collected from three independent experiments. Number of cells per group was 96 for NES PylRS/tRNA + eRF1^{E55D} + TCO*A-Lys; 58 for NES PylRS/tRNA + eRF1^{E55D}; 84 for NES PylRS/tRNA + TCO*A-Lys; 54 for NES PylRS/tRNA; 30 for Non-transfected control + TCO*A-Lys. Kruskal-Wallis non-parametric test, followed by a Mann-Whitney U test for pairwise comparisons (with Bonferroni correction for multiple comparisons) were run to compare the CTCF values of different groups. Detailed results of this statistical analysis can be found in Supplementary Table 8. The top and bottom box lines show first and third quartile of the data, while the line encompassed by the box represents the data median. Boxplot whiskers show the minimum and maximum values, with circles representing outliers. Source data are provided as a Source Data file. Scale bars: 20 µm (**a**,**b**).

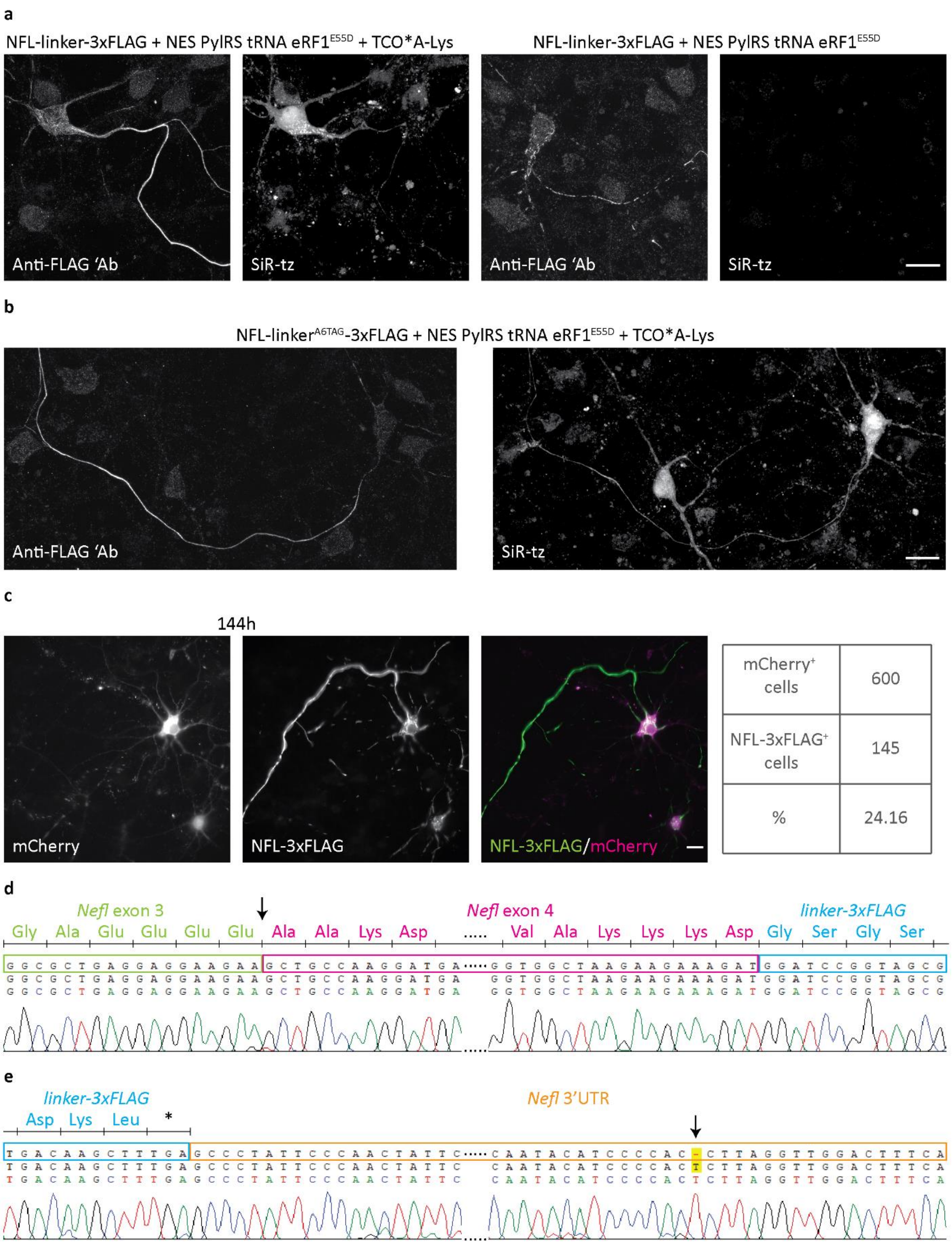

Supplementary Fig. 17 | Optimization of targeted knock-in with two guides (TKIT) approach for labeling of endogenous NFL via amber codon suppression and click chemistry. a–b, MCNs were transfected at

DIV5 with a plasmid encoding two guide RNAs (gRNAs) and Cas9, a plasmid encoding NES PylRS/$tRNA_{CUA}^{Pyl}$ and $eRF1^{E55D}$, as well as with plasmids bearing donor sequences with either $linker^{WT}$-3xFLAG (**a**) or $linker^{A6TAG}$-3xFLAG (**b**). After incubation for 6 days with or without TCO*A-Lys, neurons were labeled with SiR-tz, fixed and stained with anti-FLAG antibody, followed by AF488-conjugated secondary antibody. *Z*-stack images were acquired on a confocal scanning microscope and are shown as maximum intensity projections. Data were collected from three independent experiments. **c**, Quantification of TKIT-mediated knock-in efficiency. MCNs were transfected at DIV5 with a plasmid encoding two gRNAs and Cas9, as well as with a pcDNA3.1/Zeo(+)-mCherry plasmid bearing $linker^{WT}$-3xFLAG donor sequence. Neurons were fixed after 144 h and stained with anti-FLAG antibody, followed by AF488-conjugated secondary antibody. Panels show representative widefield images of $mCherry^+$ and NFL-$3xFLAG^+$ cells that were used for quantification. Numbers of $mCherry^+$ and NFL-$3xFLAG^+$ as well as the percentage of NFL-$3xFLAG^+$/$mCherry^+$ cells collected across two experiments are shown in the table. **d-e**, Analysis of the specificity of TKIT-mediated knock-in and *Nefl* mRNA splicing after the donor integration. MCNs were transfected at DIV5 with a plasmid encoding two gRNAs and Cas9, as well as with a pcDNA3.1/Zeo(+) plasmid bearing $linker^{WT}$-3xFLAG donor sequence. After 6 days, total RNA was extracted from neurons and used as a template for cDNA synthesis. Splice junction between *Nefl* exons 3 and 4 as well as the 3’ junction of the integrated donor DNA were amplified by PCR. Sequencing of the splice junction (**d**) and the 3’ integration junction (**e**) show that the splicing between exons 3 and 4 proceeds normally after donor integration and that the donor sequence is integrated correctly in the genomic DNA. Single nucleotide insertion, a common consequence of genome editing, located at the junction site in 3’ UTR is depicted in yellow. Arrows in **d-e** annotate the junction positions. Source data are provided as a Source Data file. Scale bars: 20 µm (**a–c**).

Supplementary Tables

Supplementary Table 1. Complete data set that was used for the western blot analysis of NFL-FLAG WT and TAG mutant expression levels in ND7/23 cells

Supplementary Table 2. Complete data set that was used for the western blot analysis of FLAG-NFL WT and TAG mutant expression levels in ND7/23 cells

Supplementary Table 3. Primers used for cloning and mutagenesis

Supplementary table 4. Donor sequences for ORANGE- and TKIT-based knock-in and primers used for PCR amplification and sequencing of genomic DNA and cDNA

Supplementary Table 5. Background values used for the deconvolution of confocal and STED images

Supplementary Table 6. Pairwise comparison of SiR-tz fluorescence background by using the Mann-Whitney U Test

Supplementary Table 7. Pairwise comparison of BODIPY-tz fluorescence background by using the Mann-Whitney U Test

Supplementary Table 8. Pairwise comparison of nonspecific click labeling background in the presence or absence of eRF1^{E55D} by using the Mann-Whitney U Test

Supplementary Table 1. Complete data set that was used for the Western blot analysis of NFL-FLAG WT and TAG mutant expression levels in ND7/23 cells

Sample	eRF^{E55D}	% of the NFL^{WT}				SEM
		WB01	WB02	WB03	Average	
NFL^{WT}-FLAG	-	100	100	100	100	0
	+	89.0	74.7	82.8	82.2	3.4
$NFL^{K211TAG}$-FLAG	-	35.9	27.6	24.8	29.4	2.7
	+	36.1	33.4	44.2	37.9	2.6
$NFL^{K363TAG}$-FLAG	-	44.1	38.6	36.6	39.8	1.8
	+	55.7	40.6	51.2	49.2	3.7
$NFL^{R438TAG}$-FLAG	-	42.6	28.0	36.3	35.6	3.5
	+	53.2	34.9	52.5	46.9	4.9
$NFL^{K468TAG}$-FLAG	-	27.9	20.8	24.9	24.6	1.7
	+	22.1	20.9	40.8	28.0	5.3

*image from NFL-FLAG WB01 is shown in the Supplementary Fig. 2a

Supplementary Table 2. Complete data set that was used for the Western blot analysis of FLAG-NFL WT and TAG mutant expression levels in ND7/23 cells

Sample		$eRF1^{E55D}$	% of the total $FLAG\text{-}NFL^{TAG}$			
			WB01	WB02	WB03	Average
$FLAG\text{-}NFL^{K211TAG}$	Full-length	-	100.0	89.1	100.0	96.4
	Fragment**	-	0.0	10.9	0.0	3.6
	Full-length	+	100.0	95.2	100.0	98.4
	Fragment	+	0.0	4.8	0.0	1.6
$FLAG\text{-}NFL^{K363TAG}$	Full-length	-	38.1	37.0	30.5	35.2
	Fragment	-	61.9	63.0	69.5	64.8
	Full-length	+	86.5	79.2	73.9	79.9
	Fragment	+	13.5	20.8	26.1	20.1
$FLAG\text{-}NFL^{R438TAG}$	Full-length	-	2.3	4.6	2.7	3.2
	Fragment	-	97.7	95.4	97.3	96.8
	Full-length	+	20.3	29.9	20.2	23.5
	Fragment	+	79.7	70.1	79.8	76.5
$FLAG\text{-}NFL^{K468TAG}$	Full-length	-	3.0	2.6	1.9	2.5
	Fragment	-	97.0	97.4	98.1	97.5
	Full-length	+	19.2	34.7	21.8	25.2
	Fragment	+	80.8	65.3	78.2	74.8

*image from FLAG-NFL WB02 is shown in the Supplementary Fig. 2d

**truncated fragments of $FLAG\text{-}NFL^{K211TAG}$ were detected only in the WB02

Supplementary Table 3. Primers used for cloning and mutagenesis

Purpose	Primer name	Primer sequence 5'–3'
Cloning of NFL in mEGFP-N1 plasmid	NfL_HindIII_fw	GGT GGT AGC TTC ACC ATG AGT TCG TTC GGC TAC GAT
	NfL_ApaI_rv	ACC ACC GGG CCC CAT CTT TCT TCT TAG CCA CCT GCT CC
Mutagenesis of NFL, position K211	NFL_K211TAG_fw	CTG GAG TAG CGC ATC GAC AGC CTG ATG GAC
	NFL_K211TAG_rv	GAT GCG CTA CTC CAG CTC GGC GCG
Mutagenesis of NFL, position K363	NFL_K363TAG_fw	AGC ACG TAG AGC GAG ATG GCC AGG TAC CT
	NFL_K363TAG_rv	CTC GCT CTA CGT GCT TCT CAG CTC ATT CTC CAG T
Mutagenesis of NFL, position R438	NFL_R438TAG_fw	TCT GCT TAG TCT TTC CCA GCC TAC TAT ACC AGC CA
	NFL_R438TAG_rv	GAA AGA CTA AGC AGA CAT CAA GTA GGA GCT GCT
Mutagenesis of NFL, position K468	NFL_K468TAG_fw	GAG GCC TAG GAT GAG CCC CCC TCT GAA GGA
	NFL_K468TAG_rv	CTC ATC CTA GGC CTC CTC AGC TTT CGT AGC
Cloning of NFL^{WT}-FLAG and NFL^{TAG}-FLAG	BamHI_FLAG_NotI_fw	GA TCC G GAC TAC AAA GAC GAT GAC GAC AAG TGA GC
	BamHI_FLAG_NotI_rv	GGC CGC TCA CTT GTC GTC ATC GTC TTT GTA GTC CG
Cloning of FLAG-NFL^{TAG} (FLAG sequence highlighted in bold)	HindIII_FLAG_NFL_fw	ACGTCCAAGCTTCACCATG**GACTACAAAGACGATGACGACAAG**AGTTCGTTCGGCTACGATCCGTAC
	NFL_STOP_NotI_rv	AGCTATGCGGCCGCTCAATCTTTCTTCTTAGCCACCTGCTCCTCT
Cloning of U6-tRNA$_{CUA}^{Pyl}$ in pcDNA3.1/Zeo(+)_NES PylRS^{AF} plasmid	U6-tRNAcassette_BglIIfw	GGTGGT AGATCT AAA AAA CGG AAA CCC CGG GAA TCT AAC C
	startU6cassette_MfeIrv	CCC TTT CAA TTG GAG GGC CTA TTT CCC ATG ATT CCT TCA TAT TTG
Cloning of pORANGE Tubb3-GFP^{Y39TAG} KI	Tubb3-GFP-KI_HindIII_fw	GGA GGA AAG CTT GCT GCG AGC AAC TTC ACT TGG GGG ATC AGG CGT GAG CAA GGG CGA GGA GC
	Tubb3-GFP-KI_XhoI_rv	TCC TCC CTC GAG CCC AAG TGA AGT TGC TCG CAG CAC ATT ACT TGT ACA GCT CGT CCA TGC CGA G
Cloning of NFL target sequence into the pORANGE cloning template vector	NFL_gRNA_BbsI_fw	CACC GAG TGC TGG AGA GGA GCA GG
	NFL_gRNA_BbsI_rv	AAA C CCT GCT CCT CTC CAG CAC TC
Cloning of linker-3xFLAG and linker^{A6TAG}-3xFLAG donor sequences in pORANGE NfL KI plasmid	NFL-link-3xFLAG KI_HindIII_fw	GGA GGA AAG CTT CC ACC TGC TCC TCT CCA GCA CTC GGT AGC GCT GGA AGC GCT
	NFL-link(A6)-3xFLAG KI_HindIII_fw	GGA GGA AAG CTT CC ACC TGC TCC TCT CCA GCA CTC GGT AGC GCT GGA AGC TAG GAC TAC
	NFL-link-3xFLAG KI_BamHI_rv	TCC TCC GGA TCC GAG TGC TGG AGA GGA GCA GGT GGT TAC TTG TCA TCG TCA TCC TTG TAA TCG ATG TCA TG
Cloning of TKIT gRNA1 and gRNA2 into the pORANGE cloning template vector	TKIT_NfLgRNA1_BbsI_fw	CACC GAC ATA TCC TTT AGG AGA GTG
	TKIT_NfLgRNA1_BbsI_rv	AAA C CAC TCT CCT AAA GGA TAT GTC
	TKIT_NfLgRNA2_BbsI_fw	CACC GTA TAA TTC TGA GAT GAC TT
	TKIT_NfLgRNA2_BbsI_rv	AAAC AA GTC ATC TCA GAA TTA TAC
Cloning of U6 promoter-gRNA2 cassette into the TKIT U6 promoter-gRNA1 construct	XbaI_U6_fw	GGAGGA TCT AGA GTG AGG GCC TAT TTC CCA TGA TTC CTT C
	SalI_gRNA scaffold_rv	TCC TCC GTC GAC GAA TTG GCG CAC GCG CTA AAA AC

Supplementary table 4. Donor sequences for ORANGE- and TKIT-based knock-in and primers used for PCR amplification and sequencing of genomic DNA and cDNA

Purpose	Donor/primer name	Sequence 5'–3'
Donor DNA for ORANGE-based knock-in (position of the linker A6 codon highlighted in bold)	Donor sequence with linker^{WT}-3xFLAG	CCACCTGCTCCTCTCCAGCACTCGGTAGCGCTGGAAGC**GCT**G<br>ACTACAAAGACCATGACGGTGATTATAAAGATCATGACATCG<br>ATTACAAGGATGACGATGACAAGTAACCACCTGCTCCTCTCC<br>AGC ACT C
	Donor sequence with linker^{A6TAG}-3xFLAG	CCACCTGCTCCTCTCCAGCACTCGGTAGCGCTGGAAGC**TAG**G<br>ACTACAAAGACCATGACGGTGATTATAAAGATCATGACATCG<br>ATTACAAGGATGACGATGACAAGTAACCACCTGCTCCTCTCC<br>AGC ACT C
Donor DNA for TKIT-based knock-in (position of the linker A6 codon highlighted in bold)	Donor sequence with linker^{WT}-3xFLAG	AGATCTCCTAAGTCATCTCAGAATTATACGTGGGGCCAA<br>GAAGTTTTTAGACACAGTCTGACAATAGTGATTATTAGCCCA<br>CAAAGCCTCAGTAGAAATGGTTTGCCTAGACCTACCCTG<br>GAGTTTTGTATTTTCTTTTTATGTTCAAGCTGCCAAGGATGA<br>GTCTGAAGACACAAAAGAAGAAGAAGAAGGTGGTGAGGG<br>TGAGGAGGAAGACACCAAAGAATCTGAAGAGGAAGAGAA<br>GAAAGAGGAGAGTGCTGGAGAGGAGCAGGTGGCTAAGAA<br>GAAAGATGGATCCGGTAGCGCTGGAAGC**GCT**GACTACAA<br>AGACCATGACGGTGATTATAAAGATCATGACATCGATTA<br>CAAGGATGACGATGACAAGCTTTGAGCCCTATTCCCAACTAT<br>TCCAGGAAAAGTTCTCCCCAATCAGGTCAACCTCATCACCAA<br>CCAACCAGTTGAGTTCCAGATCCTATACAAATTAAGAAGTCA<br>ATACATCCCCACTCTCCTAAAGGATATGTCAATTG
	Donor sequence with linker^{A6TAG}-3xFLAG	AGATCTCCTAAGTCATCTCAGAATTATACGTGGGGCCAA<br>GAAGTTTTTAGACACAGTCTGACAATAGTGATTATTAGCCCA<br>CAAAGCCTCAGTAGAAATGGTTTGCCTAGACCTACCCTG<br>GAGTTTTGTATTTTCTTTTTATGTTCAAGCTGCCAAGGATGA<br>GTCTGAAGACACAAAAGAAGAAGAAGAAGGTGGTGAGGG<br>TGAGGAGGAAGACACCAAAGAATCTGAAGAGGAAGAGAA<br>GAAAGAGGAGAGTGCTGGAGAGGAGCAGGTGGCTAAGAA<br>GAAAGATGGATCCGGTAGCGCTGGAAGC**TAG**GACTACAA<br>AGACCATGACGGTGATTATAAAGATCATGACATCGATTA<br>CAAGGATGACGATGACAAGCTTTGAGCCCTATTCCCAACTAT<br>TCCAGGAAAAGTTCTCCCCAATCAGGTCAACCTCATCACCAA<br>CCAACCAGTTGAGTTCCAGATCCTATACAAATTAAGAAGTCA<br>ATACATCCCCACTCTCCTAAAGGATATGTCAATTG
Amplification/sequencing of the 5' donor integration site for pORANGE KI	Nefl_intron3_fw	CCT TTC AGA ATC TCT ATT GCA AAT TGG CTG TGG
	3xFLAG_rv	CTT GTC ATC GTC ATC CTT GTA ATC GAT GTC ATG ATC
Amplification/sequencing of the 3' donor integration site for pORANGE KI	linker-3xFLAG_fw	GGA AGC GCT GAC TAC AAA GAC CAT GAC
	Nefl_3'UTR_rv	CCC TCA GAA TGT CCA ATT ATC TAC TGC ACT CAC
Amplification/sequencing of the Nefl exon3-exon4 splicing site for TKIT KI	Nefl_exon3_TKIT_fw	CTC TTG GAA GGC GAA GAG ACC AGG
	BamHI-linker_rv	GCT TCC AGC GCT ACC GGA T
Amplification/sequencing of the 3' donor integration site for TKIT KI	linker-3xFLAG_fw	GGA AGC GCT GAC TAC AAA GAC CAT GAC
	Nefl_3'UTR2_rv	ATT GCC ATA GAT CCT GAA CTC ATA AGC ATG GAC

Supplementary Table 5. Background values used for the deconvolution of confocal and STED images

Figure	Image	Background value
Fig. 5a	SiR-tz confocal	5
	SiR-tz STED	0.5
Fig. 5b	SiR-tz confocal	4
	SiR-tz STED	5
	ATTO488-tz confocal	3
	ATTO488-tz STED	5
Supplementary Fig. 12a	ATTO488-tz confocal	2.5
	ATTO488-tz STED	1.5
Supplementary Fig. 12b	CF650-me-tz confocal	5
	CF650-me-tz STED	1.5
Supplementary Fig. 12c	SiR-tz confocal	2
	SiR-tz STED	2
Supplementary Fig. 13	SiR-tz confocal	5
	SiR-tz STED	2
	ATTO488-tz confocal	3
	ATTO488-tz STED	4
Supplementary Fig. 14b	SiR-tz STED	1
	ATTO488-tz STED	4
Supplementary Fig. 14c	SiR-tz STED	3
	ATTO488-tz STED	4

Supplementary Table 6. Pairwise comparison of SiR-tz fluorescence background by using the Mann-Whitney U Test

Comparison #	Groups†	N	Mean Rank	Sum of ranks	U	Z	P (2-tailed)
1	1	30	45.50	1356.00	0.000	-6.653	0.000*
	2	30	15.50	465.00			
2	1	30	39.67	1190.00	175.00	-4.066	0.000*
	3	30	21.33	640.00			
3	1	30	40.07	1202.00	163.00	-4.243	0.000*
	4	30	20.93	628.00			
4	1	30	45.50	1365.00	0.000	-6.653	0.000*
	5	30	15.50	465.00			
5	1	30	41.97	1259.00	106.00	-5.086	0.000*
	6	30	19.03	571.00			
6	2	30	15.53	466.00	1.000	-6.638	0.000*
	3	30	45.47	1364.00			
7	2	30	15.50	465.00	0.000	-6.653	0.000*
	4	30	45.50	1365.00			
8	2	30	31.70	951.00	414.00	-0.532	0.595
	5	30	29.30	879.00			
9	2	30	15.57	467.00	2.000	-6.623	0.000*
	6	30	45.43	1363.00			
10	3	30	27.73	832.00	367.00	-1.227	0.220
	4	30	33.27	998.00			
11	3	30	45.50	1365.00	0.000	-6.653	0.000*
	5	30	15.50	465.00			
12	3	30	30.67	920.00	445.00	-0.074	0.941
	6	30	30.33	910.00			
13	4	30	45.50	1365.00	0.000	-6.653	0.000*
	5	30	15.50	465.00			
14	4	30	33.77	1013.00	352.00	-1.449	0.147
	6	30	27.23	817.00			
15	5	30	15.60	468.00	3.000	-6.609	0.000*
	6	30	45.40	1362.00			

*indicates significance (with corrected Bonferroni $p < 0.0033$ for 15 comparisons)

†Groups: 1. TCO*A-Lys + 2 h washing; 2. – UAA + 2h washing; 3. endo-BCN-Lys + 2 h washing; 4. TCO*A-Lys + 10 h washing; 5. – UAA + 10 h washing; 6. endo-BCN-Lys + 10 h washing

Supplementary Table 7. Pairwise comparison of BODIPY-tz fluorescence background by using the Mann-Whitney U Test

Comparison #	Groups†	N	Mean Rank	Sum of ranks	U	Z	P (2-tailed)
1	1	30	45.50	1365.00	0.000	-6.653	0.000*
	2	30	15.50	465.00			
2	1	30	40.93	1228.00	137.00	-4.628	0.000*
	3	30	20.07	602.00			
3	1	30	42.10	1263.00	102.00	-5.145	0.000*
	4	30	18.90	567.00			
4	1	30	45.50	1365.00	0.000	-6.653	0.000*
	5	30	15.50	465.00			
5	1	30	43.17	1295.00	70.00	-5.618	0.000*
	6	30	17.83	535.00			
6	2	30	15.50	465.00	0.000	-6.653	0.000*
	3	30	45.50	1365.00			
7	2	30	15.50	465.00	0.000	-6.653	0.000*
	4	30	45.50	1365.00			
8	2	30	39.40	1182.00	183.00	-3.947	0.000*
	5	30	21.60	648.00			
9	2	30	16.10	483.00	18.00	-6.387	0.000*
	6	30	44.90	1374.00			
10	3	30	33.63	1009.00	356.00	-1.390	0.165
	4	30	27.37	821.00			
11	3	30	45.50	1365.00	0.000	-6.653	0.000*
	5	30	15.50	465.00			
12	3	30	32.80	984.00	381.00	-1.020	0.308
	6	30	28.20	846.00			
13	4	30	45.50	1365.00	0.000	-6.653	0.000*
	5	30	15.50	465.00			
14	4	30	29.90	897.00	432.00	-0.266	0.790
	6	30	31.10	933.00			
15	5	30	15.60	468.00	3.000	-6.609	0.000*
	6	30	45.40	1362.00			

*indicates significance (with corrected Bonferroni $p < 0.0033$ for 15 comparisons)

†Groups: 1. TCO*A-Lys + 2 h washing; 2. – UAA + 2h washing; 3. endo-BCN-Lys + 2 h washing; 4. TCO*A-Lys + 10 h washing; 5. – UAA + 10 h washing; 6. endo-BCN-Lys + 10 h washing

Supplementary Table 8. Pairwise comparison of nonspecific click labeling background in the presence or absence of eRF1[E55D] by using the Mann-Whitney U Test

Comparison #	Groups†	N	Mean Rank	Sum of ranks	U	Z	P (2-tailed)
1	1	96	104.19	10002.00	222.00	-9.553	0.000*
	2	58	33.33	1933.00			
2	1	96	84.79	8140.00	3484.00	-1.571	0.116
	3	84	97.02	8150.00			
3	1	96	100.93	9689.00	151.00	-9.557	0.000*
	4	54	30.30	1636.00			
4	1	96	76.11	7307.00	229.00	-6.936	0.000*
	5	30	23.13	694.00			
5	2	58	31.03	1800.00	89.00	-9.741	0.000*
	3	84	99.44	8353.00			
6	2	58	62.10	3602.00	1241.00	-1.892	0.058
	4	54	50.48	2726.00			
7	2	58	36.64	2125.00	414.00	-4.014	0.000*
	5	30	59.70	1791.00			
8	3	84	95.86	8052.00	54.00	-9.659	0.000*
	4	54	28.50	1539.00			
9	3	84	71.48	6004.00	86.00	-7.555	0.000*
	5	30	18.37	551.00			
10	4	54	32.87	1775.00	290.00	-4.854	0.000*
	5	30	59.83	1795.00			

*indicates significance (with corrected Bonferroni $p < 0.005$ for 10 comparisons)

†Groups: 1. NES PylRS/tRNA + eRF1[E55D] + TCO*A-Lys; 2. NES PylRS/tRNA + eRF1[E55D]; 3. NES PylRS/tRNA + TCO*A-Lys; 4. NES PylRS/tRNA; 5. Non-transfected + TCO*A-Lys

Source Data files

Supplementary Fig. 2a

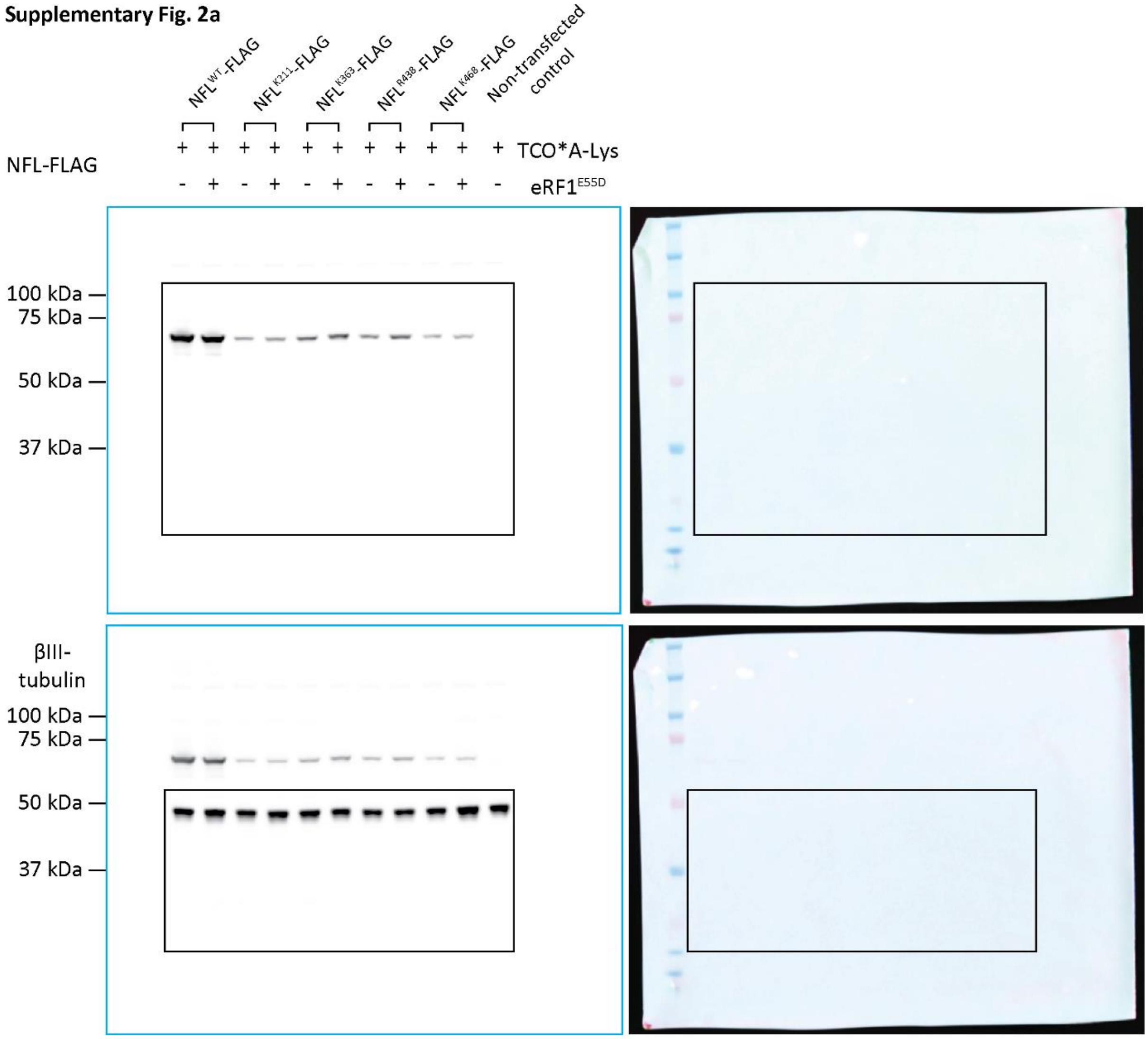

Supplementary Fig. 2b

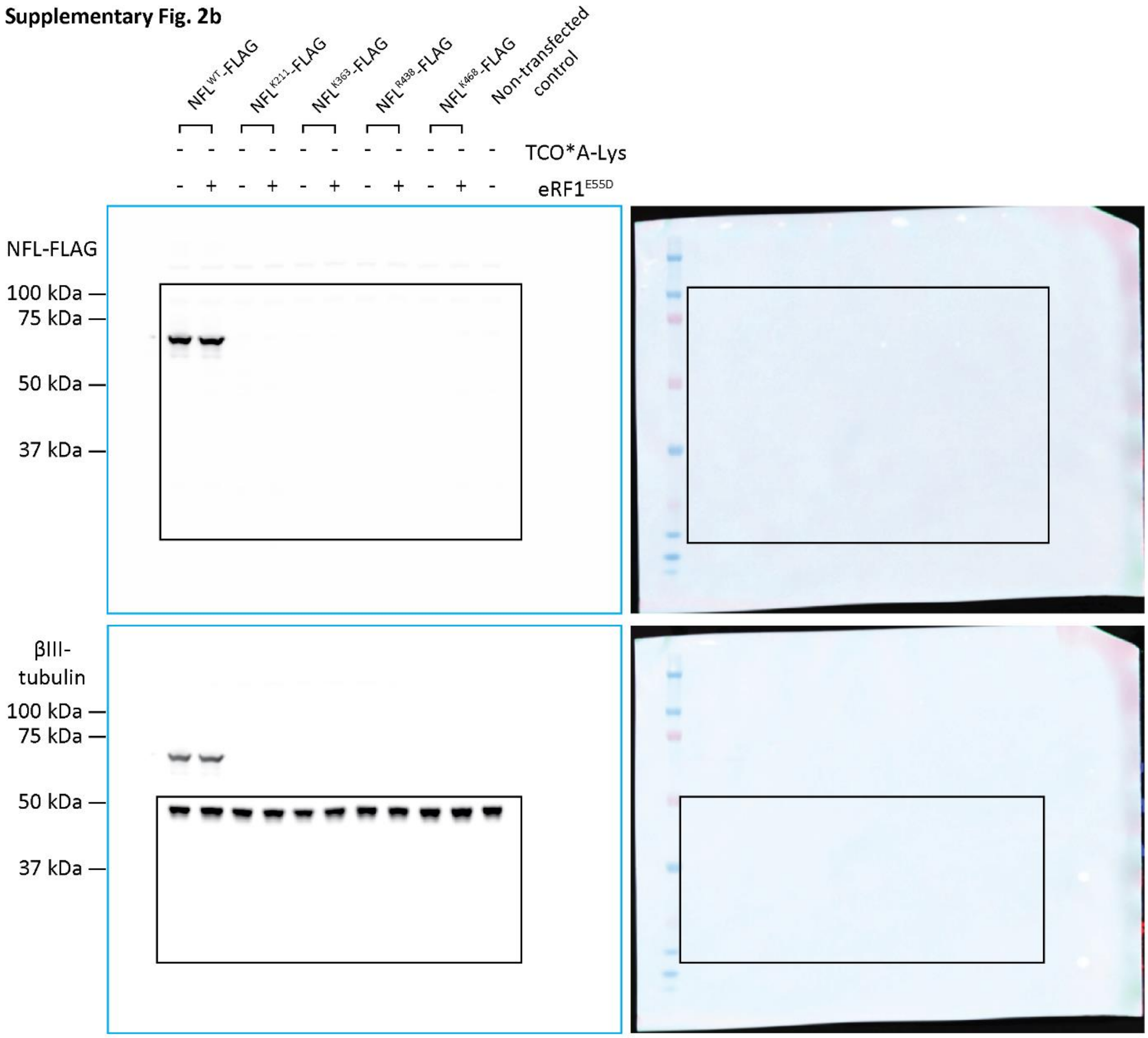

Supplementary Fig. 2d

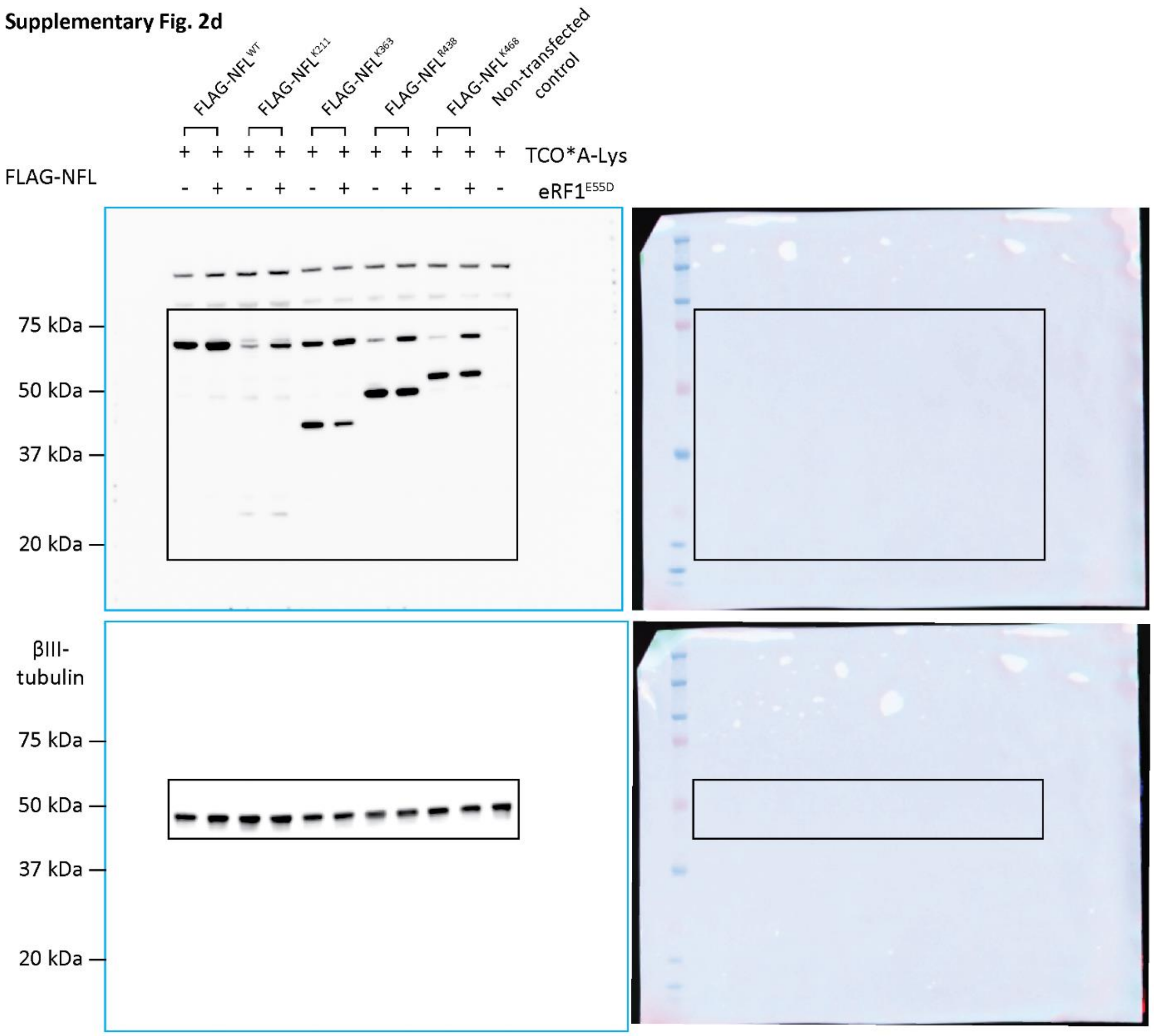

Supplementary Fig. 2e

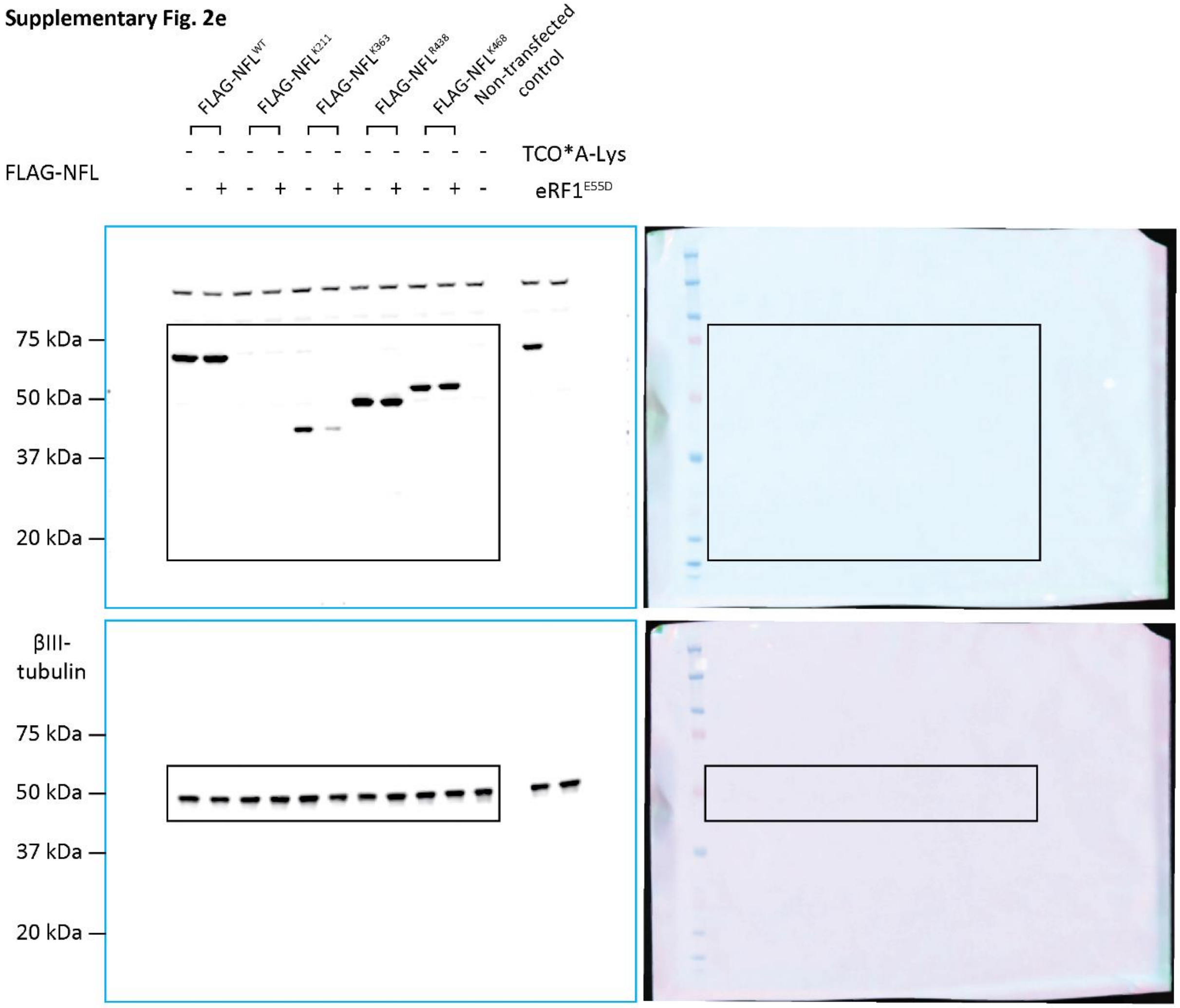

Supplementary Fig. 3a

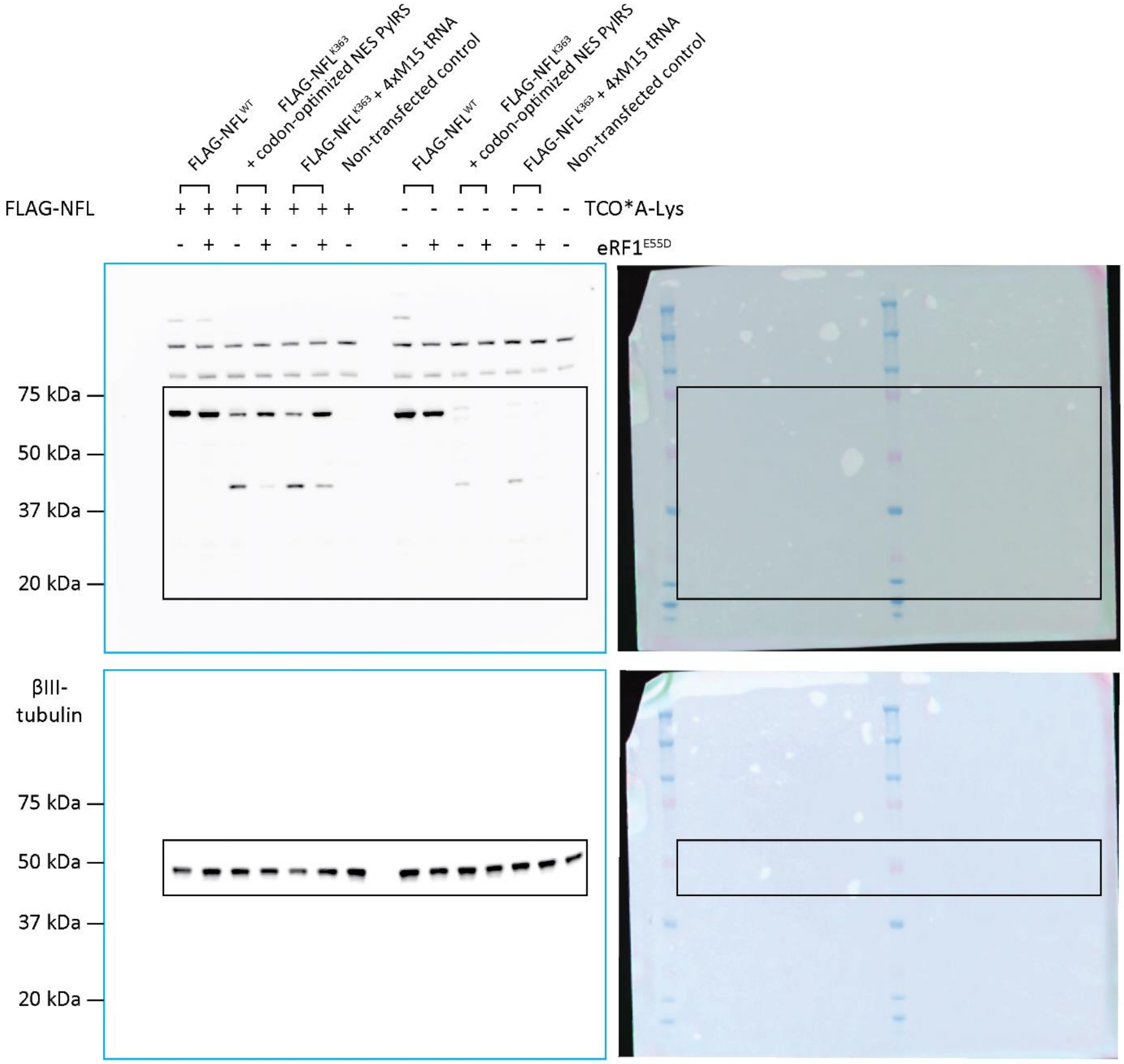

Supplement: Supplementary file 1 — Supplementary Information [file 41467_2022_27956_MOESM1_ESM.pdf]
