# Supplementary material for: Minimal genetically encoded tags for fluorescent protein labeling in living neurons
Source: Nat Commun. 2022 Jan 14;13:314. doi: 10.1038/s41467-022-27956-y (PMC8760255; doi:10.1038/s41467-022-27956-y)
Supplement: Supplementary file 2 — Reporting Summary [file 41467_2022_27956_MOESM2_ESM.pdf]

nature research

Corresponding author(s): Ivana Nikić-Spiegel

Last updated by author(s): Dec 14, 2021

Reporting Summary

Nature Research wishes to improve the reproducibility of the work that we publish. This form provides structure for consistency and transparency in reporting. For further information on Nature Research policies, see our Editorial Policies and the Editorial Policy Checklist.

Statistics

For all statistical analyses, confirm that the following items are present in the figure legend, table legend, main text, or Methods section.

n/a	Confirmed	
☐	☒	The exact sample size (*n*) for each experimental group/condition, given as a discrete number and unit of measurement
☐	☒	A statement on whether measurements were taken from distinct samples or whether the same sample was measured repeatedly
☐	☒	The statistical test(s) used AND whether they are one- or two-sided<br>*Only common tests should be described solely by name; describe more complex techniques in the Methods section.*
☒	☐	A description of all covariates tested
☐	☒	A description of any assumptions or corrections, such as tests of normality and adjustment for multiple comparisons
☒	☐	A full description of the statistical parameters including central tendency (e.g. means) or other basic estimates (e.g. regression coefficient) AND variation (e.g. standard deviation) or associated estimates of uncertainty (e.g. confidence intervals)
☐	☒	For null hypothesis testing, the test statistic (e.g. *F*, *t*, *r*) with confidence intervals, effect sizes, degrees of freedom and *P* value noted<br>*Give P values as exact values whenever suitable.*
☒	☐	For Bayesian analysis, information on the choice of priors and Markov chain Monte Carlo settings
☒	☐	For hierarchical and complex designs, identification of the appropriate level for tests and full reporting of outcomes
☐	☒	Estimates of effect sizes (e.g. Cohen's *d*, Pearson's *r*), indicating how they were calculated

Our web collection on statistics for biologists contains articles on many of the points above.

Software and code

Policy information about availability of computer code

Data collection	Images were acquired on commercial microscopes as described in the Methods section, using NIS-Elements AR 4.60 (Nikon Instruments), ZEN 2011 SP7 FP3 14.0.19.201 (Zeiss) and LAS X 3.5.5.19976 (Leica) software. Chemiluminescence for Western blot was visualized using Azure 600 imager (Azure Biosystems).
Data analysis	All images were analyzed in Fiji/ImageJ 1.53c. For presentation purposes, all images were converted to 8-bit depth using Fiji and arranged into figures using Adobe Illustrator CS5 (version 15.0.0). The schemes presented in the manuscript were made using the BioRender app (www.BioRender.com) and Adobe Illustrator CS5 (version 15.0.0).<br>STED images and the corresponding confocal images were deconvolved using Huygens deconvolution software version 17.10.0p6 64b (SVI, Netherlands).<br>Western blot analysis was done using AzureSpot software version 1.6.2.1020 (Azure Biosystems).<br>Statistical analysis was done in IBM SPSS Statistics VS25.<br>Sequencing analysis was done using the Vector NTI Advance software version 11.5.4 (Life Technologies).

For manuscripts utilizing custom algorithms or software that are central to the research but not yet described in published literature, software must be made available to editors and reviewers. We strongly encourage code deposition in a community repository (e.g. GitHub). See the Nature Research guidelines for submitting code & software for further information.

Data

Policy information about availability of data

All manuscripts must include a data availability statement. This statement should provide the following information, where applicable:

- Accession codes, unique identifiers, or web links for publicly available datasets
- A list of figures that have associated raw data
- A description of any restrictions on data availability

All the data supporting the results are provided in the manuscript or supplementary information. Raw microscopy images are available on Figshare under the following DOI: 10.6084/m9.figshare.c.5749409. Source data are provided with this paper. Plasmids generated in this study are available on request (via material transfer agreement) from the corresponding author or will be made available on Addgene. Publicly available dataset Ensembl, release 102, November 2020; Mus musculus version 102.38 (GRCm38.p6; http://nov2020.archive.ensembl.org/Mus_musculus/Info/Index?db=core) was used for the selection of guide sequences for CRISPR/Cas9 genome engineering.

Field-specific reporting

Please select the one below that is the best fit for your research. If you are not sure, read the appropriate sections before making your selection.

- [x] Life sciences
- [ ] Behavioural & social sciences
- [ ] Ecological, evolutionary & environmental sciences

For a reference copy of the document with all sections, see nature.com/documents/nr-reporting-summary-flat.pdf

Life sciences study design

All studies must disclose on these points even when the disclosure is negative.

Sample size	No statistical method was used to predetermine the sample size. We determined the sample size based on published work as will be described below.<br>For the experiments involving click labeling background quantification shown in Supplementary Figure 10, we acquired and analyzed following number of images: 10 images per condition per experiment x 3 experiments = 30 images per condition x 6 conditions = 180 images for SiR-tetrazine and 180 images for BDPtz. In total, we acquired 360 images and we analyzed all images. Previously, for similar type of analysis, Uttamapinant et al., (https://doi.org/10.1021/ja512838z) and Kozma et al., (https://doi.org/10.1002/cbic.201600284) analyzed around 15 cells per timepoint. Since we managed to obtain more than 15 cells per condition in our dataset, we considered this number to be sufficient.<br>For the experiments involving click labeling background quantification +/- eRF1(E55D) shown in Supplementary Figure 16c, we acquired images of all transfected neurons across three experiments. This resulted in: 96 images for NES PylRS/tRNA + eRF1(E55D) + TCO*A-Lys; 84 images for NES PylRS/tRNA + TCO*A-Lys; 58 images for NES PylRS/tRNA + eRF1(E55D); 54 images for NES PylRS/tRNA; 30 images for Non-transfected control + TCO*A-Lys. This equals to 323 images in total. We analyzed them all, except for one image in which the neuron looked dead.<br>For the experiments involving ORANGE efficiency quantification shown in Supplementary Fig. 15e, we decided to analyze at least 500 transfected (mCherry+) neurons per timepoint. To this aim, we acquired images of all transfected neurons across three experiments. In total, 1780 images were acquired across three experiments. We analyzed 534 neurons per timepoint (178 neurons per experiment per timepoint). In the original article describing this method (Wilems et al., 2020 https://doi.org/10.1371/journal.pbio.3000665), ORANGE efficiency was estimated by analyzing around 1000 transfected neurons from two independent neuronal cultures. We analyzed less neurons per experiment but did three experiments which overall we thought was sufficient.<br>For the experiments involving quantification of TKIT efficiency shown in Supplementary Fig. 17c, we decided to count 300 transfected (mCherry +) neurons per experiment. To this aim, we acquired images of all transfected neurons across two experiments. In total, 627 images were acquired. The total number of analyzed neurons was 600. In the original article describing this method (Fang et al., eLife 2021;10:e65202 doi: 10.7554/eLife.65202) TKIT efficiency was estimated by analyzing around 150-300 neurons collected across 3-4 experiments. We analyzed higher number of neurons than previous study and we considered it sufficient.
Data exclusions	No data was excluded.
Replication	Each experiment (except for anti-FLAG immunostaining control shown in Supplementary Fig. 6c,d and TKIT incorporation efficiency shown in Supplementary Fig. 17c) was repeated at least three times. Replicated experiments were successful. Control anti-FLAG immunostaining and colocalization analysis as shown in Supplementary Fig. 6c,d was performed twice, but the same antibody was reproducibly used for anti-FLAG staining in other experiments. TKIT incorporation efficiency experiments were done twice. As described above in the section about the sample size, we analyzed significantly higher number of neurons compared to the literature (Fang et al., 2021 analyzed 150-300 neurons across 3-4 experiments and we analyzed 600 neurons across 2 experiments) and that is the reason why we did only two experiments.
Randomization	Cells and neurons were randomly allocated into different experimental groups. This was done for all biological and technical replicates. To limit bias, for experiments involving comparisons of different conditions (treatments), experiments and data collection were performed in parallel. This was done for all techniques, including microscopy and western blots.
Blinding	Blinding during data collection was not performed since the same investigator did the group allocation (cell/neuron seeding, transfections, click labeling etc) and the data collection. Furthermore, blinding during data collection was not feasible since the investigator had to adjust microscopy settings to fit with different fluorophore combinations. Analysis of the data presented in plots in Supplementary Figures 10 and 16c was done in a blinded way. Analysis of the data presented in Supplementary Figures 15e comparing efficiency of ORANGE knock-in at different timepoints (24h, 72h, 144h) was not done in the blinded way since we know from the publish work (Wilems et al., 2020 https://doi.org/10.1371/journal.pbio.3000665) that higher number of neurons will be transfected at later days post-transfection. This was obvious during data collection and that is why the counting of mCherry+/FLAG+ neurons was not done in the blinded way. Blinding was not relevant to

the analysis of the data presented in Supplementary Fig. 17c since it involved only one timepoint.

Reporting for specific materials, systems and methods

We require information from authors about some types of materials, experimental systems and methods used in many studies. Here, indicate whether each material, system or method listed is relevant to your study. If you are not sure if a list item applies to your research, read the appropriate section before selecting a response.

Materials & experimental systems

n/a	Involved in the study
☐	☒ Antibodies
☐	☒ Eukaryotic cell lines
☒	☐ Palaeontology and archaeology
☒	☐ Animals and other organisms
☒	☐ Human research participants
☒	☐ Clinical data
☒	☐ Dual use research of concern

Methods

n/a	Involved in the study
☒	☐ ChIP-seq
☒	☐ Flow cytometry
☒	☐ MRI-based neuroimaging

Antibodies

Antibodies used

Neurofilament 70s kDa (mouse, Merck Millipore, cat. no. MAB1615, lot no. 3209184, clone DA2)
Anti-FLAG antibody (rabbit, Merck Millipore cat. no. F7425, lot no. 078M4886V)
Goat anti-rabbit Alexa Fluor (AF) 488 Plus (Thermo Fisher Scientific, cat. no. A32731)
Goat anti-rabbit AF555 (Thermo Fisher Scientific, cat. no. A21429)
Goat anti-mouse AF555 (Thermo Fisher Scientific, cat. no. A21424)
Goat anti-rabbit AF647 Plus (Thermo Fisher Scientific, cat. no. A32733)
Goat anti-mouse AF647 Plus (Thermo Fisher Scientific, cat. no. A32728)
Mouse anti-FLAG M2 antibody (Sigma-Aldrich, cat.no. F1804)
Mouse anti-βIII tubulin antibody (BioLegend, cat. no. 801202)
Goat anti-mouse AF488 Plus (Thermo Fisher Scientific, cat. no. A32723)
Goat anti rabbit horseradish peroxidase (HRP; Thermo Fisher Scientific, cat. no. A16104)
Goat anti-mouse HRP (Thermo Fisher Scientific, cat. no. A16072).

Validation

All antibodies were purchased from commercial sources, and validated by the data sheets of the manufacturer or citations listed on manufacturer's websites:
Neurofilament 70 kDa (mouse, Merck Millipore, cat. no. MAB1615, lot no. 2965867, clone DA2): more than 45 references are listed on the manufacturer’s website. According to the manufacturer, the antibody can be used for WB/ICC/IHC, has following species reactivity (bovine, human, mouse, pig, rat), and is routinely tested on human cerebellum tissue. No further information is provided, and we could confirm its reactivity in mouse neurons.
Anti-FLAG antibody (rabbit, Sigma/Merck Millipore cat. no. F7425, lot no. 078M4886V): more than 2192 references are listed on the manufacturer’s website. According to the manufacturer, the antibody can be used for following applications: dot blot: 1-2.5 μg/mL
immunoprecipitation (IP): 4-8 μg using amino terminal FLAG-BAP fusion protein from E. coli crude lysate
indirect immunofluorescence: 5-10 μg/mL using 293T cells transfected with a plasmid encoding FLAG-JNK
western blot (chemiluminescent): 1-2.5 μg/mL using an E. coli periplasmic extract expressing an N-terminal FLAG fusion protein and is reactive against all species. No further information is provided.
Mouse anti-FLAG M2 antibody (Sigma-Aldrich, cat.no. F1804): more than 6489 references are listed on the manufacturer’s website. According to the manufacturer, the antibody can be used for following applications: For highly sensitive and specific detection of FLAG fusion proteins by immunoblotting, immunoprecipitation (IP) , immunohistochemisty, immunofluorescence and immunocyotchemistry. Optimized for single banded detection of FLAG fusion proteins in mammalian, plant, and bacterial expression systems. Western Blotting and EIA. And is reactive against all species.
Mouse anti-βIII tubulin antibody (BioLegend, cat. no. 801202): more than 597 references are listed on the manufacturer’s website. According to the manufacturer, the antibody is reactive against human, mouse, rat. And can be used for following applications: IHC-P - Quality tested; WB, ICC - Verified; FC - Reported in the literature, not verified in house. No further information is provided.

Eukaryotic cell lines

Policy information about cell lines

Cell line source(s)

ND7/23 cell line: mouse neuroblastoma x rat neuron hybrid. The cell line was obtained from Sigma Aldrich (ECACC 92090903).

Authentication

ND7/23 cell line was authenticated by their providers. Immunostainings for two neuronal markers (tubulin β3 and neurofilament light chain) performed in our laboratory also confirm its neuronal origin.

Mycoplasma contamination

ND7/23 cell line was confirmed negative for mycoplasma contamination by their provider.

Commonly misidentified lines<br>(See ICLAC register)	ND7/23 cell line is not in the ICLAC register of commonly misidentified lines.
